# CRISPR Screening Reveals a Novel Role for FOXH1 in Regulating Pluripotency of Porcine Embryonic Stem Cells

**DOI:** 10.1002/advs.202509495

**Published:** 2025-07-11

**Authors:** Peng Su, Linhui Wu, Delong Li, Wenting Song, Dagang Tao, Liang Liu, Qi Wang, Manxin Gao, Tian Xu, Xin Liu, Shengsong Xie, Xia Zhang, Jilong Zhou, Yi‐Liang Miao

**Affiliations:** ^1^ Institute of Stem Cell and Regenerative Biology College of Animal Science and Veterinary Medicine Huazhong Agricultural University Wuhan 430070 P. R. China; ^2^ Key Laboratory of Agricultural Animal Genetics Breeding and Reproduction Huazhong Agricultural University Ministry of Education Wuhan 430070 P. R. China; ^3^ Hubei Hongshan Laboratory Wuhan 430070 P. R. China; ^4^ Frontiers Science Center for Animal Breeding and Sustainable Production Huazhong Agricultural University Ministry of Education Wuhan 430070 P. R. China

**Keywords:** CRISPR‐Cas9 screening, FOXH1, pluripotency, porcine extended potential stem cells (pEPSCs), self‐renewal

## Abstract

Porcine extended potential stem cells (pEPSCs), which exhibit both self‐renewal and pluripotency, are promising for application in both agricultural biotechnology and regenerative medicine. However, the molecular mechanisms governing these two interconnected properties remain elusive. Here, two types of CRISPR‐Cas9 screenings are conducted in pEPSCs. This fitness screening identified several genes essential for cell viability, including *PRMT1*, *MYBL2*, and *NASP*. Concurrently, FACS‐based screenings revealed genes crucial for pluripotency, such as *SOX2*, *ZFP42*, and *FOXH1*. Notably, it is demonstrated that FOXH1 is required for maintaining pluripotency in pEPSCs, which complements the understanding of its role in mesendoderm specification. pEPSCs lacking FOXH1 exhibited a flatter and more dispersed clonal morphology, accompanied by downregulation of pluripotency genes and upregulation of lineage‐specific genes. Additionally, *FOXH1* knockdown significantly impaired blastocyst formation during early pig embryogenesis. Functionally, the dual role of FOXH1 in pluripotency maintenance and cell differentiation is validated: FOXH1 preserves pluripotency by enhancing chromatin accessibility at pluripotency gene loci, while also influencing lineage specification through H3K4me3 modification at developmental related genes. Thus, these findings uncover a novel role of FOXH1 involved in the core regulatory network that orchestrates gene expression programs to maintain the pluripotency state of pEPSCs and provide valuable insights into categorizing gene function.

## Graphical abstract

This study performs dual CRISPR screens in pEPSCs. Fitness screening identifies essential genes for viability, while FACS‐based screening uncovers core pluripotency factors. It highlights FOXH1 as a key regulator that maintains the stem cell state through a dual‐action mechanism: preserving pluripotency by enhancing chromatin accessibility and influencing lineage specification via epigenetic modification. These findings provide critical species‐specific insights.

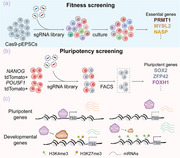

## 1 Introduction

Embryonic stem cells (ESCs) are the in vitro extension of the developing embryo, with the ability to differentiate into all somatic tissues.^[^ ^1^ ^]^ Recently, the establishment of ESCs in animal livestock species, particularly porcine ESCs (pESCs), has shown promising solutions to challenges such as transgenic pig production, xenotransplatation and animal breeding.^[^ ^2^, ^3^, ^4^ ^]^ Although significant efforts have been made to obtain the bona fide pESCs based on the pluripotency criteria established for human and mouse ESCs,^[^ ^5^, ^6^, ^7^ ^]^ naive state pESCs have not yet been established. This is partly because the development of pig stem cell lines has largely followed models based on human and mouse ESCs, while pESCs display unique molecular characteristics and exhibit distinct dependency on signaling pathways like Activin and Wnt.^[^ ^8^ ^]^ As a result, the molecular regulatory mechanisms underlying their pluripotency need further investigation.

The maintenance of self‐renewal and pluripotency of pESCs is governed by a delicate balance within a pluripotency regulatory network. This network safeguards the viability and proliferation of pESCs in vitro, supporting cell fitness, alongside preserves the pluripotent identity. The regulation mechanisms have been extensively explored, involving multiple transcription factors, epigenetic factors, and exogenous signaling pathways.^[^ ^9^, ^10^, ^11^ ^]^ For instance, POU5F1^[^ ^12^, ^13^ ^]^ and NANOG^[^ ^14^, ^15^ ^]^ are recognized as master regulators of pluripotent identity and variation in their expression levels directly influence cell fate decisions. They simultaneously regulate each other while cooperatively orchestrate the expression of a broad array of downstream genes to sustain the pluripotent state.^[^ ^16^, ^17^, ^18^ ^]^ While self‐renewal and pluripotency regulatory systems display universal features, there is also emerging evidence of specific regulatory pathways.^[^ ^19^ ^]^ However, how to distinguish and identify the specific genes that regulate self‐renewal and pluripotency in pESCs remains a significant challenge.

Functional genomics screening represents a robust and unbiased approach for delineating the roles of uncharacterized genes. The genome‐wide RNAi or shRNA screening methods have been gradually abandoned due to some intrinsic technical drawbacks such as the incomplete degradation of proteins, confounding off‐target effects, and the risk of reporting false positives associated.^[^ ^20^ ^]^ The CRISPR‐Cas9 system has been widely used to screen for essential genes and therapeutic targets in various cell types, owing to its ability to precisely knock out genes at the DNA level.^[^ ^21^, ^22^, ^23^, ^24^, ^25^ ^]^ Despite significant progress in the field, the use of whole‐genome CRISPR‐Cas9 screenings to explore the mechanisms of self‐renewal and pluripotency in pESCs is still unreported. This is due to the high costs and complex requirements of pESC culture^[^ ^5^ ^]^ and the lower efficiency of genetic manipulation.^[^ ^26^ ^]^ Moreover, screenings focused on cell fitness might overlook other crucial pluripotency regulators with only minor or moderate effects on survival and proliferation.^[^ ^27^ ^]^ To address these issues, we designed two types of CRISPR‐Cas9 knockout screenings to independently investigate the genes that control cell fitness and those that regulate pluripotent identity in pEPSCs, using a pooled CRISPR library targeting all annotated transcription factors (TFs) and epigenetic regulators.

In this study, our fitness screening identified 900 essential genes. Among these, the conserved genes across species, such as *PRMT1*, *MYBL2*, and *PCNA*, along with the porcine‐specific genes *NASP* and *FOS*, were experimentally validated at the cellular level as critical for preserving the proliferation of pEPSCs. Subsequently, during the screening focused on pluripotency, we discovered new targets such as the transcription factor FOXH1 (FAST1, forkhead activin signal transducer 1), revealing its novel function in pluripotency regulation and further expanding the core pluripotency network involving POU5F1 and NANOG. Overall, our two types of CRISPR screenings provide an efficient framework for gene identification and functional annotation, further provide unique insights into pluripotency regulation in pESCs, and establish a theoretical foundation for more extensive CRISPR screenings of pESCs under other culture conditions.

## 2 Results

### 2.1 Identification of Essential Genes in pEPSC

Before conducting CRISPR library screening, we successfully derived pEPSCs from pre‐implantation blastocysts of Bama porcine in vivo using pEPSC culture medium (Figure S1A and Table S1, Supporting Information).^[^ ^5^ ^]^ The pEPSCs were passaged every 3–4 days as single cells at a 1:3 ratio through enzymatic dissociation. Throughout long‐term culture, the pEPSCs retained positive alkaline phosphatase (AP) staining and normal karyotypes (Figure S1B,C, Supporting Information). Moreover, immunofluorescent staining showed that high‐passage pEPSCs continued to express core pluripotency markers (Figure S1D, Supporting Information). To further perform sgRNA screening, we generated a Cas9‐expressing cell line via lentiviral infection, which was confirmed by Western blot (Figure S1E,F, Supporting Information).

**Figure 1:**
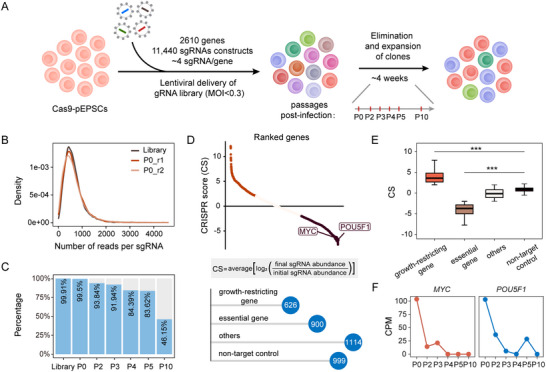
Establishment of a loss‐of‐function library to identify essential genes in pEPSCs. A) Schematic overview of the fitness screening. B) Density distribution of read counts per sgRNA for the initial plasmid library (Library) and the initial mutant cell library (P0, which includes two replications). C) Histogram of sgRNA library coverage analysis comparing the initial plasmid library (Library) to subsequent passages of mutation cell libraries following sgRNA viral transduction. A noticeable decrease in sgRNA coverage is evident as the culture passages increase. The blue columns indicate the measured sgRNA with counts exceeding 1, while the grey columns represent the missing sgRNA. D) Ranked all genes in the final mutant cell library by CRISPR score (CS), which is calculated as the average log_2_ fold‐change in the abundance of all sgRNAs targeting each gene. Bottom panel: Genes categorized by CS into different types: growth‐ restricting genes, essential genes, and other genes, with the number of genes in each category displayed. E) Comparison of CS for four types of genes, showing significant differences between essential genes and non‐target control as well as between growth‐ restricting genes and non‐target control. ^***^ *p* < 0.001; two‐tailed Student's t test. F) Reduction in sgRNA abundance for essential genes *MYC* and *POU5F1* during the screening process. The x‐axis shows different time points (P0, P2, P3, P4, P5, P10) during the screening.

We designed and constructed a rapid and efficient CRISPR‐Cas9 loss‐of‐function screening targeting all 2610 annotated TFs and epigenetic regulators in the porcine genome according to a previous study (**Figure** **1**A; Table S2, Supporting Information).^[^ ^24^ ^]^ The CRISPR library comprises 4 single‐guide RNAs (sgRNAs) per gene, along with 1000 non‐target control sgRNAs (NTCs) that are predicted not to target any genomic loci in pigs. Cas9‐expressing pEPSCs were infected with lentivirus containing this library. To minimize the risk of multiple sgRNAs integrating into a single cell, we adopted a low multiplicity of infection (MOI) to achieve a transduction rate of less than 30%, based on previous studies.^[^ ^24^, ^28^ ^]^ Comparison between the pre‐transfection plasmid library and the initial cell library post‐puromycin selection (p0) showed a consistent distribution of sgRNA reads, with two independent transductions demonstrating good reproducibility (Figure 1B). Following selection with puromycin, we cultured and passaged the cells for 30 days, collecting genomic DNA samples at multiple time points for deep sequencing. The sequencing volumes of sgRNAs in the cell library at different time points were similar, which aids in the comparison across different passages (Figure S2A, Supporting Information). As expected, the CRISPR library demonstrated a progressive decline in sgRNA representation and a corresponding reduction in sgRNA library diversity over time (Figure 1C; Figure S2B, Supporting Information). This decline was driven by the selective depletion of cells harboring mutations in genes essential for survival and pluripotency, resulting in notable sgRNA losses by the final time point (Figure S2C, Supporting Information). During the screening process, the correlation coefficient between sgRNA reads in cell libraries from successive generations and the initial cell library notably diminished (Figure S2D, Supporting Information). This was accompanied by a gradual increase in the count of sgRNAs that were completely lost (Figure S2E, Supporting Information), indicating a dynamic shift in sgRNA composition over successive generations.

To analyze the significant changes in sgRNA expression between the initial and final populations of pEPSCs, we devised a CRISPR Score^[^ ^29^ ^]^ to rank genes. This metric represents the ratio of sgRNA abundance across the final versus initial populations for each gene. Based on this analysis, we identified 900 essential genes and 626 growth‐restricting genes, distinguished by the loss and enrichment of associated sgRNAs, respectively (Figure 1D). Relative to NTCs, essential genes exhibited significantly lower CRISPR Scores, whereas growth‐restricting genes displayed higher scores (Figure 1E). Notably, these identified essential genes include *POU5F1* ^[^ ^30^ ^]^ and *MYC* ^[^ ^31^ ^]^ which are crucial for the survival of ESCs and demonstrated a consistent downward trend during the screening process (Figure 1F). This result underscores the effectiveness of our screening method. Additionally, these essential genes are uniformly dispersed across all chromosomes, with no specific enrichment in distinct chromosomal regions (Figure S2F, Supporting Information).

**Figure 2:**
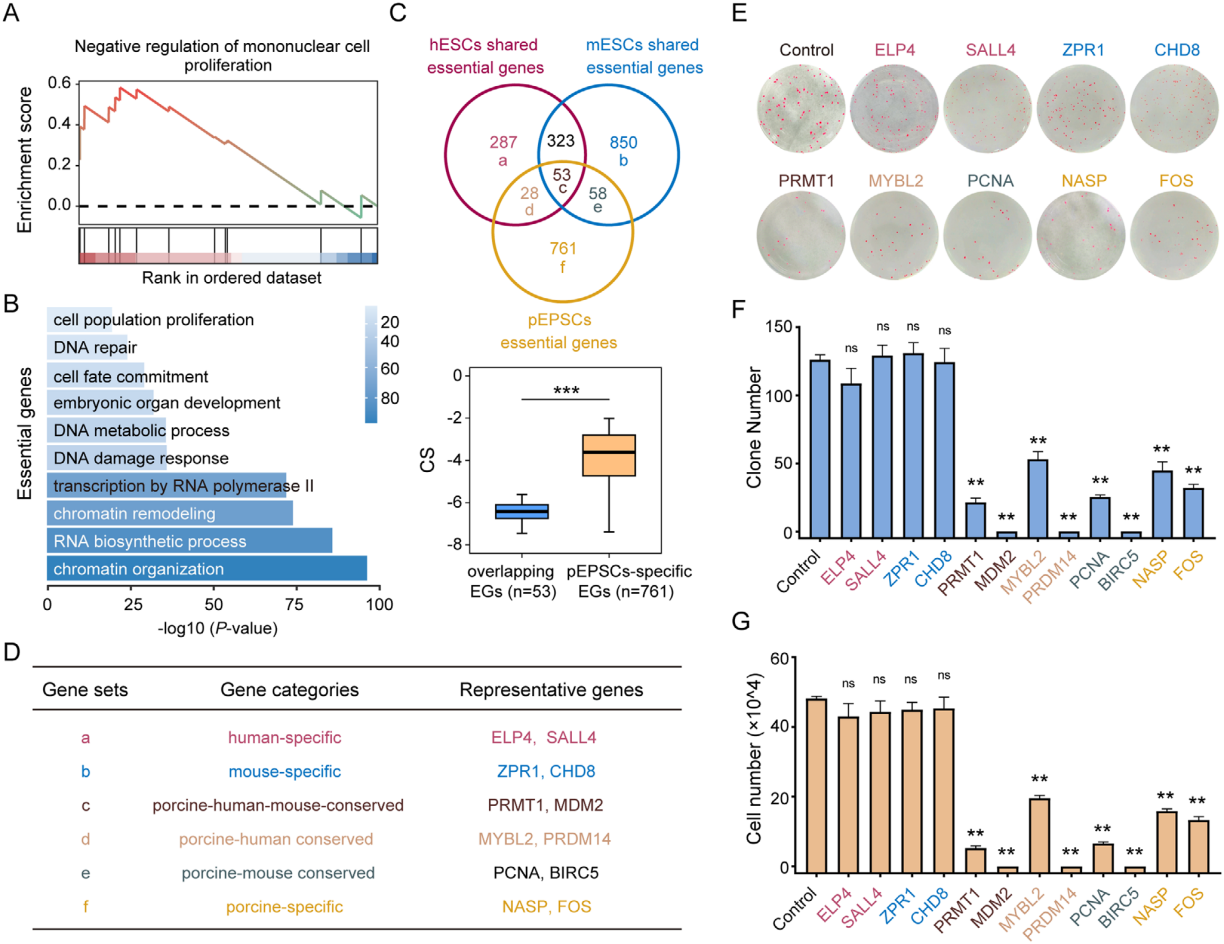
Characterization and validation of essential genes identified in pEPSCs. A) GSEA pathway analysis enrichment plot shows enrichment of growth‐ restricting genes. B) GO enrichment analysis of essential genes. C) Venn diagram showing the overlap of essential genes identified in pEPSCs in the current study with those shared by hESCs and mESCs. Bottom panel: CS of overlapping EGs with pEPSC‐specific EGs. D) Representative genes across different gene sets. E,F) Colony formation assay featuring AP staining (E) and quantification of colony numbers F), comparing non‐target control with knockout of target genes. G) The indicated pEPSCs (10000 cells per well in 12‐well plates) were cultured for 5 days, and cell numbers were counted to assess the proliferation ability of non‐target control and target gene knockout cells.Data in (F,G) are represented as the mean ± SD; *n* = 3 independent experiments; ns, non‐significant; ^*^ *p* < 0.05; ^**^ *p* < 0.01; two‐tailed Student's t test.

To elucidate the functional roles of genes identified through our screening, we initially performed a Gene Set Enrichment Analysis (GSEA) that revealed a significant enrichment of growth‐restricting genes within pathways negatively regulating proliferation (**Figure** **2**A). Additionally, Gene Ontology (GO) analysis of essential genes highlighted their enrichment in core cellular processes crucial for cell survival, including chromatin organization, RNA biosynthesis, DNA metabolism, response to DNA damage, cell fate commitment, and population growth (Figure 2B). Considering the potential limitations of a single CRISPR library or screening conditions, we expanded our analysis by comparing our dataset with those derived from previous screenings in human and mouse ESCs (Figure 2C; Figure S2G and Table S3, Supporting Information).^[^ ^22^, ^32^, ^33^, ^34^ ^]^ Intriguingly, this comparison analysis revealed 53 conserved essential genes across species and 761 genes uniquely essential to pEPSCs. To confirm the accuracy of the screening and integrative analysis, we selected genes from various defined gene sets to further explore their roles in the self‐renewal of pEPSCs (Figure 2D). Many of these genes have been previously identified as essential in hESCs or mESCs.^[^ ^22^, ^32^, ^33^, ^34^ ^]^ Interestingly, knockouts of species‐specific genes (human and mouse) in pEPSCs, such as *ELP4*, *SALL4*, *ZPR1*, and *CHD8*, did not compromise cell survival. In contrast, knockouts of genes that are conserved across species or specific to porcine, such as *PRMT1*, *NASP*, *MDM2*, *FOS*, *PRDM14*, *MYBL2*, *PCNA*, and *BIRC5*, resulted in a significant decrease in colony formation and proliferation compared to control cells transduced with NTC sgRNAs (Figure 2E–G). Notably, the knockouts of *MDM2*, *PRDM14*, and *BIRC5* directly resulted in cell death. These results collectively suggest that both the conserved across species and porcine‐specific genes are essential and valid for normal growth in pEPSCs, while essential genes specific to other species might not be required.

### 2.2 CRISPR Screening Identifies Regulators of Pluripotency

Regulators of pluripotency do not invariably promote the survival of stem cells. Screening focused on survival can potentially overlook other crucial pluripotency regulators that apply gentle or moderate influences on cellular proliferation. To identify these pluripotency regulators, we used POU5F1 and NANOG as markers to assess the pluripotent state because they are key indicators of core pluripotency and early embryogenesis. By employing CRISPR‐Cas9 Knock‐in technology, we generated two pluripotency reporter cell lines: *NANOG*‐tdTomato and *POU5F1*‐tdTomato (Figure S3A and Table S2, Supporting Information). To assess the sensitivity of these reporter lines to changes in gene expression, we performed gene knockdown (KD) experiments using *NANOG* siRNA and *POU5F1* siRNA, respectively (Figure S3B, Supporting Information). Following gene knockdown, we observed a marked diminution in tdTomato fluorescence intensity in two reporter lines (Figure S3C, Supporting Information). Further functional validation was undertaken using a differentiation culture system^[^ ^35^ ^]^ that facilitated the downregulation of pluripotency‐associated genes and the upregulation of markers for the three germ layers (Figure S3D, Supporting Information). During the differentiation process, pEPSC clones became flatter and their boundaries less distinct. Concurrently, both fluorescence microscopy and flow cytometry revealed a gradual decrease in the intensity and overall proportion of tdTomato fluorescence in the *NANOG* and *POU5F1* reporter cell lines (Figure S3E,F, Supporting Information). Taken together, these findings suggest that these reporter cell lines are useful for mirroring the downregulation of NANOG and POU5F1 expression as the pluripotency network disintegrates.

**Figure 3:**
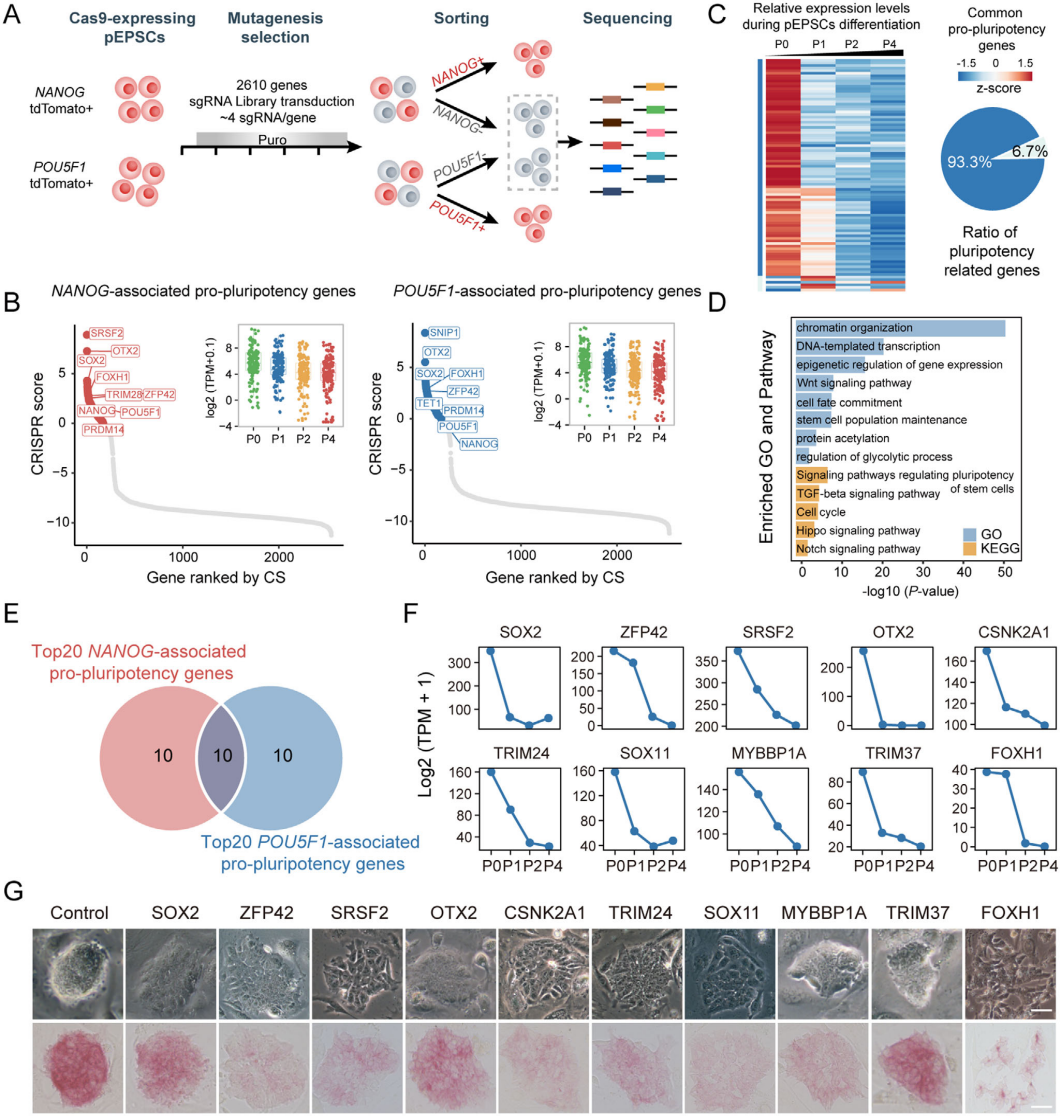
NANOG and POU5F1 FACS‐based screenings identify pro‐pluripotency genes in pEPSCs. A) The workflow of the screenings using *NANOG*‐tdTomato and *POU5F1*‐tdTomato pluripotency reporter cell lines. Pluripotent ESCs are depicted as pink while differentiating cells as grey. B) CRISPR score distribution for pro‐pluripotency genes associated with NANOG and POU5F1, showing their expression in different passages during the differentiation process. C) Heatmap showing the dynamic expression levels of common pluripotency‐related genes (log_2_(TPM+0.1)) identified through screening with two reporter lines during differentiation passages (P0‐P4). D) GO enrichment and KEGG pathway analysis of common pluripotency‐related genes shown in Figure 3C. E) Venn diagram depicting the overlap between top 20 *NANOG*‐associated and *POU5F1*‐associated pro‐pluripotency genes. F) Expression changes of 10 candidate pro‐pluripotency genes (log_2_(TPM+1)) in different passages during the differentiation process. G) Representative morphology and AP staining of colonies with knockout of 10 candidate pro‐pluripotency genes. Scale bar, 100 µm.

Then we used these two different reporter lines for CRISPR screenings to identify the common regulatory factors essential for promoting pluripotency (**Figure** **3**A). Both reporter lines were infected with lentivirus containing our CRISPR library and cultured for 3 to 4 generations under puromycin selection. After screening, cell populations exhibiting negative or diminished tdTomato fluorescence were collected using fluorescence‐activated cell sorting (FACS) technology to obtain genomic DNA samples for deep sequencing. Similar to our first CRISPR screening, the diversity within the initial cell library remained largely consistent with that of the pre‐transfection plasmid library. However, we observed a significant reduction in sgRNA diversity post mutagenesis selection (Figure S4A, Supporting Information).

We next utilized the CRISPR Score to rank genes associated with promoting pluripotency (pro‐pluripotency genes) related to *NANOG* or *POU5F1*. Meanwhile, by integrating our previously published RNA‐seq data^[^ ^35^ ^]^ on the differentiation process of pEPSCs, we observed a general trend of gradual downregulation in these pro‐pluripotency genes during cellular differentiation (Figure 3B). Then we focused on 90 overlapping genes identified through screening with two reporter cell lines, of which 93.3% are associated with pluripotency (Figure 3C). These potential candidate genes formed a tight protein‐protein network with known pluripotency regulators, which was enriched in transcriptional regulators and chromatin‐modifying factors involved in the regulation of stem cell pluripotency (Figure S4B, Supporting Information). GO enrichment and Kyoto Encyclopedia of Genes and Genomes (KEGG) pathway analysis revealed significant enrichment of these genes in terms critical for pluripotency, such as chromatin organization, maintenance of stem cell populations, cell fate determination, and various pluripotency‐related signaling pathways (Figure 3D). In summary, these results suggest that our screenings have successfully identified positive regulatory factors of pluripotency

To further validate the role of these pluripotency regulators, we selected the top 20 genes based on CRISPR Score from each category as primary candidates. Upon overlapping analysis, we identified 10 candidate pro‐pluripotency genes (Figure 3E), including reported core pluripotency genes, such as *SOX2* ^[^ ^36^ ^]^ and *ZFP42*.^[^ ^37^ ^]^ Analysis of our previous RNA‐seq data revealed that all of these 10 genes exhibited a trend of gradually declining expression during differentiation (Figure 3F). We then infected two different pluripotency reporter cell lines with lentiviral vectors carrying gene‐target sgRNAs. The fluorescence intensity of *NANOG*‐tdTomato and *POU5F1*‐tdTomato exhibited variable reductions following the knockout (KO) of these candidate genes (Figure S4C,D, Supporting Information). Notably, *FOXH1* KO and *ZFP42* KO showed similar levels of fluorescence reduction, whereas *TRIM37* KO resulted in only a mild decrease. Additionally, we observed varying degrees of differentiation in cells following the knockout of these candidate genes (Figure 3G; Table S4, Supporting Information). For instance, there was a slight lightening in AP staining following *TRIM37* KO. In contrast, *FOXH1* KO cells displayed flatter and more dispersed clonal morphology with significantly reduced intercellular contacts. Given the most significant impact observed following the *FOXH1* KO, we decided to further investigate its potential role in maintaining pluripotency.

### 2.3 FOXH1 is Required for Maintenance of the Pluripotent State

To investigate the role of FOXH1 in pluripotency regulation while minimizing effects from other genetic manipulations, we generated *FOXH1* deficient cells using low‐passage wild‐type (WT) pEPSCs. Following single‐cell seeding for clonal expansion, the resulting clones were isolated and subjected to PCR analysis to identify those with homozygous mutations. We analyzed 12 colonies and successfully isolated one knockout clone missing 29 bp in both alleles (Figure S5A, Supporting Information). We next confirmed the absence of *FOXH1* expression in the mutated cell line through real‐time quantitative PCR (RT‐qPCR) using mutation‐specific primers and corroborated by immunofluorescence assays (Figure S5B,C, Supporting Information). In addition, a T7ENI cleavage assay confirmed the high specificity and activity of the sgRNA, and revealed no off‐target cleavage at any predicted sites (Figure S5D,E, Supporting Information). Furthermore, cell proliferation assays indicated that the knockout of *FOXH1* had minimal impact on cell viability (Figure S5F, Supporting Information). This finding further suggests that FOXH1 plays a specific role in maintaining pluripotency, independent of its effects on cell survival.

**Figure 4:**
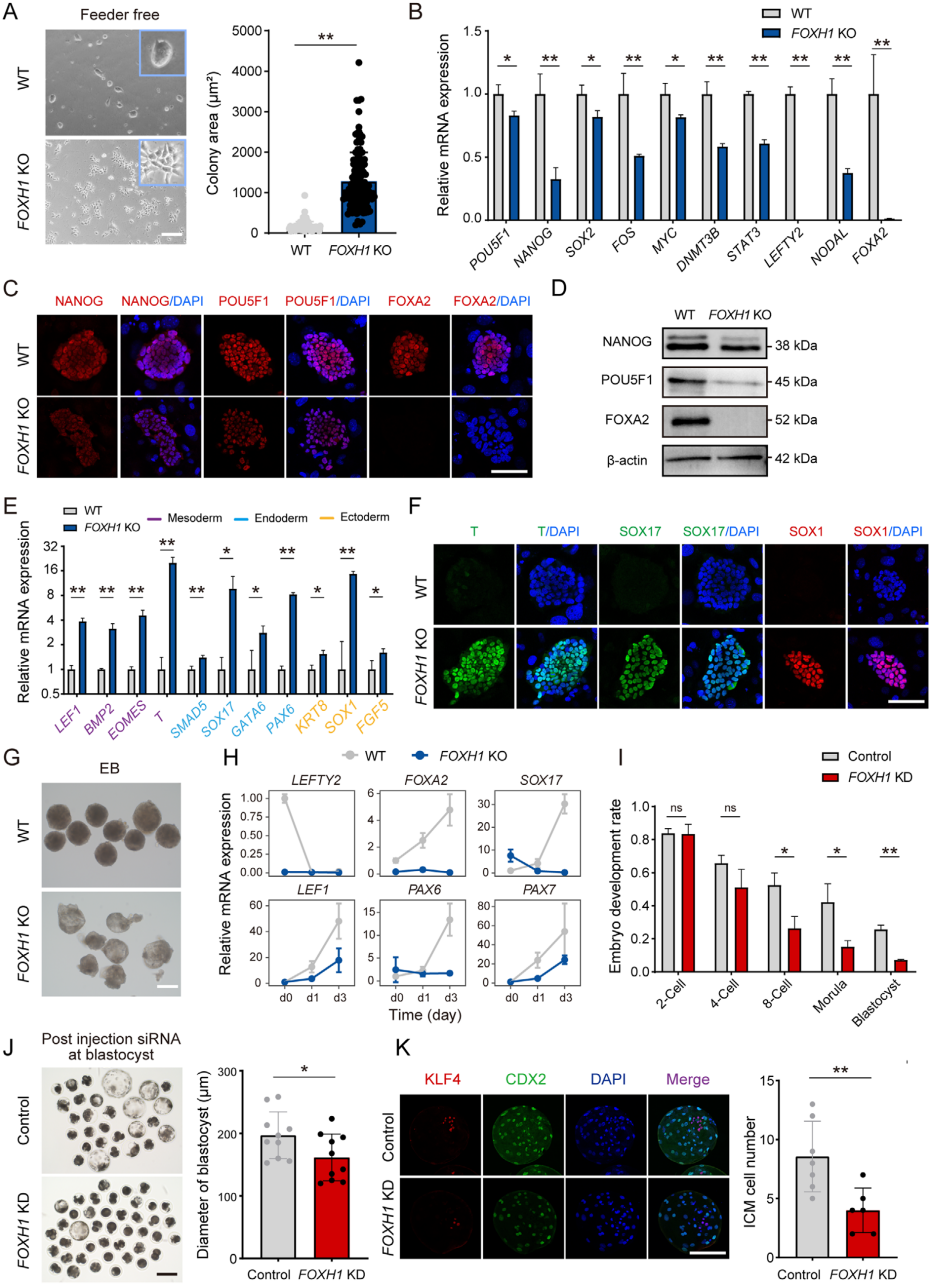
Defining the novel functions of FOXH1 in pluripotency maintenance. A) Representative morphology of WT and *FOXH1* KO pEPSCs in feeder‐free conditions and quantification of the mean colony area (± SD) for over 100 individual WT and *FOXH1* KO colonies. Scale bar, 100 µm. ^**^ *p* < 0.01; two‐tailed Student's t test. B) RT‐qPCR analysis for the expression level of pluripotency‐related genes in WT and *FOXH1* KO pEPSCs. Data are shown as mean ± SD. ^*^ *p* < 0.05; ^**^ *p* < 0.01; two‐tailed Student's t test. C) Immunofluorescence staining for NANOG, POU5F1, and FOXA2 in WT and *FOXH1* KO pEPSCs. Scale bar, 100 µm. D) Western blot analysis of NANOG, POU5F1, and FOXA2 expression in WT and *FOXH1* KO pEPSCs, utilizing β‐actin as an internal control for normalization. E) RT‐qPCR analysis for the expression levels of three germ layers gene in WT and *FOXH1* KO pEPSCs. Data are shown as mean ± SD. ^*^ *p* < 0.05; ^**^ *p* < 0.01; two‐tailed Student's t test. F) Immunofluorescence staining for T, SOX17, and SOX1 in WT and *FOXH1* KO pEPSCs. Scale bar, 100 µm. G) Representative morphology of embryoid body (EB) formation in WT and *FOXH1* KO pEPSCs. Scale bar, 100 µm. H) RT‐qPCR analysis for the expression levels of pluripotency and three germ layers markers during EB formation over three days. I) Bar charts showing the development rate of negative control and *FOXH1* KD embryos at the indicated time points post siRNA injection. The numbers of embryos analyzed from three independent experiments are shown. Data are shown as mean ± SD. ^*^ *p* < 0.05; ^**^ *p *< 0.01; two‐tailed Student's t test. J) Representative morphology of blastocyst development following injection of negative control and *FOXH1* siRNA at the zygotic stage, cultured for 6.5 days in vitro. Scale bar, 100 µm. Right panel: Quantification of blastocyst diameter in negative control and *FOXH1* KD embryos. Data are shown as mean ± SD. ^*^ *p* < 0.05; two‐tailed Student's t test. K) Co‐immunofluorescence staining for KLF4 and CDX2 in negative control and *FOXH1* KD blastocysts. Scale bar, 100 µm. Right panel: Quantification of ICM cell number in negative control and *FOXH1* KD blastocysts. Data are shown as mean ± SD. ^**^ *p *< 0.01; two‐tailed Student's t test.

Given our previous observations of distinct behaviors between WT and *FOXH1* KO cells in culture, we systematically quantified these differences by culturing both cell lines under feeder‐free conditions for two passages prior to initiating differentiation. In the absence of feeder cells, WT pEPSCs predominantly formed smaller, dome‐shaped colonies typical of pluripotent stem cells, whereas *FOXH1* KO colonies tended to be flatter and covered more of the culture surface area (**Figure** **4**A). RT‐qPCR and immunofluorescence staining revealed that *FOXH1* KO leads to a significant reduction in both RNA and protein levels of the core pluripotency factors POU5F1 and NANOG (Figure 4B,C; Table S4, Supporting Information). This observation was further supported by Western blot (Figure 4D). These findings align with above pro‐pluripotency screenings for pluripotency regulators. Moreover, expression levels of other pluripotency‐associated genes such as *SOX2*, *DNMT3B*, *STAT3*, *LEFTY2* and cell‐cell adhesion modulators^[^ ^38^ ^]^ like *NODAL* and *FOXA2* were notably decreased (Figure 4B–D; Table S4, Supporting Information), whereas markers of the three germ layers including *T*, *LEF1*, *SOX17*, *GATA6*, *SOX1*, *KRT8* were significantly elevated (Figure 4E,F; Figure S5G,H and Table S4, Supporting Information). These results indicate an inability of pEPSCs to sustain pluripotency in the absence of FOXH1.

We next sought to determine whether *FOXH1* KO pEPSCs retain the potential to differentiate into any somatic cell type. Embryoid body (EB) differentiation assays showed that WT EBs were uniform in shape and size, presenting compact, well‐defined spherical structures. Conversely, *FOXH1* KO EBs displayed considerable morphological irregularities, with unclear boundaries and instances of fragmentation (Figure 4G). After more than three days, the EBs formed by knockout cells no longer survived. The RT‐qPCR analysis revealed that during differentiation, the expression of three germ layer markers such as *SOX17*, *LEF1*, and *PAX6* was slower in *FOXH1* KO compared to WT EBs (Figure 4H; Table S4, Supporting Information), suggesting potential disruptions in cellular organization, differentiation, or developmental processes under FOXH1 deficient conditions.

Next, we investigated whether the observed phenotype of differentiating cells reflect embryonic functions of FOXH1 during early embryonic development. Initially, we characterized *FOXH1* expression during early development in porcine and human embryos using reported RNA‐seq datasets.^[^ ^7^, ^39^, ^40^ ^]^ *FOXH1* expression begins in the morula stage of porcine embryos and continues through to the post‐late‐epiblast stage, whereas in humans, *FOXH1* is highly expressed starting from the late‐blastocyst stage (Figure S5I, Supporting Information). The differences in the timing of expression might lead to different cellular phenotypes in hPSCs and pEPSCs when FOXH1 is absent.^[^ ^32^, ^41^ ^]^ We then employed siRNA‐mediated knockdown of *FOXH1* mRNA in embryos, using embryos treated with nontargeting siRNA as a negative control. The interference efficiency of *FOXH1*, which exceeded 95%, was validated via RT‐qPCR at the morula stage (Figure S5J, Supporting Information). This knockdown resulted in a significant reduction in the rate of blastocyst formation, with only 7.2% of embryos developing to blastocysts in the KD group compared to 25.7% in the control group (Figure 4I). Additionally, *FOXH1* knockdown caused a marked reduction in blastocyst diameter, indicative of compromised blastocyst quality (Figure 4J). We subsequently utilized KLF4 and CDX2 immunostaining to distinguish inner cell mass (ICM) and trophectoderm (TE) in the blastocysts (Figure 4K). CDX2 specifically labels the TE, whereas KLF4 marks the ICM. Statistical analysis revealed a substantial decrease in the number of KLF4‐positive cells in the KD group relative to controls, suggesting that *FOXH1* interference may lead to alterations in cell fate. The significant decrease in KLF4‐positive cells underscores potential deficiencies in the viability or developmental competence of the ICM in KD group blastocysts. Taken together, these findings highlight the novel role of FOXH1 in pluripotent cells and its critical impact on the developmental potential of blastocysts.

### 2.4 FOXH1 Maintains an Open Chromatin of its Target Pluripotency‐Associated Gene

**Figure 5:**
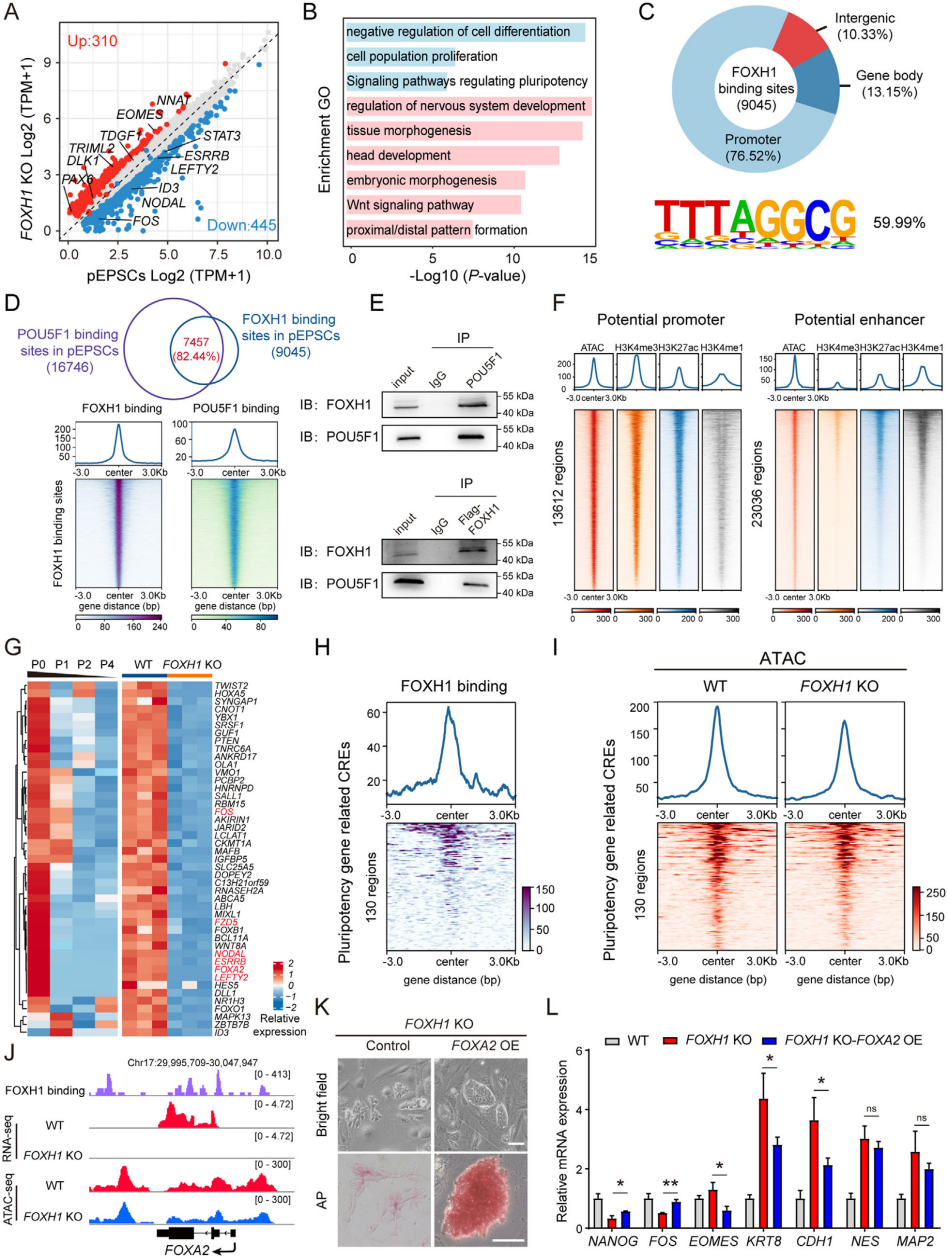
FOXH1 maintains open chromatin of its target pluripotency genes. A) Volcano plot showing down‐ (log_2_FC < ‐1, adjusted *p* < 0.05) and upregulated (log_2_FC > 1, adjusted *p* < 0.05) genes in WT and *FOXH1* KO pEPSCs. B) GO enrichment analysis of genes downregulated and upregulated following *FOXH1* KO. C) Distribution of FOXH1 binding sites in the porcine genome, with the binding motif shown below. D) Venn diagram showing overlap between FOXH1 and POU5F1 binding sites in pEPSCs, with heatmaps illustrating the distribution of binding intensity across gene regions. E) Co‐immunoprecipitation (CO‐IP) detection of FOXH1 interacting with POU5F1. Up: Endogenous POU5F1 in pEPSCs was immunoprecipitated with a POU5F1 antibody, followed by Western blot detection of FOXH1 and POU5F1. Down: Endogenous FOXH1 in pEPSCs was immunoprecipitated with Flag‐conjugated agarose beads, followed by Western blot detection of FOXH1 and POU5F1. F) Heatmaps displaying identified CREs, including potential promoters and potential enhancers. Active promoters are identified by H3K4me3 enrichment and high chromatin accessibility. Active enhancers are characterized by the co‐enrichment of H3K4me1 and H3K27ac, along with open chromatin states. G) Heatmaps showing changes in expression of potential FOXH1 target genes associated with pluripotency, based on published RNA‐seq data. H) ChIP‐seq profiles of FOXH1 binding at pluripotency‐related CREs. The signal in a ± 3 kb window flanking the peak center is shown. I) ATAC‐seq profiles showing chromatin accessibility in WT and *FOXH1* KO conditions at pluripotency‐related CREs bound by FOXH1. The signal in a ± 3 kb window flanking the peak center is shown. J) The IGV snapshot shows ATAC‐seq and RNA‐seq of the CRE regions of FOXA2 in WT and *FOXH1* KO pEPSCs. K) Representative morphology and AP staining of *FOXH1* KO cells and overexpressed *FOXA2* based on *FOXH1* KO cells. Scale bars, 100 µm. L) RT‐qPCR analysis for the expression levels of pluripotency markers and lineage genes in *FOXH1* KO and overexpressed *FOXA2* based on *FOXH1* KO cells. Data are shown as mean ± SD. ns, non‐significant; ^*^ *p* < 0.05; ^**^ *p* < 0.01; two‐tailed Student's t test.

To better understand the molecular function of FOXH1 in pEPSCs, we conducted RNA‐seq with *FOXH1* KO and WT pEPSCs. Setting a threshold for significant changes at an expression fold‐change greater than 1 and a *p*‐value below 0.05 (false discovery rate [FDR] < 0.1), we used DE‐seq2 to identify 310 genes with increased expression and 445 genes with decreased expression in *FOXH1* KO cells (**Figure** **5**A; Table S5, Supporting Information). Notably, GO analysis highlighted genes downregulated in *FOXH1* KO cells were significantly enriched for terms such as negative regulation of cell differentiation, signaling pathways regulating pluripotency, and others. In contrast, upregulated genes in *FOXH1* KO cells were predominantly associated with cell differentiation GO terms, which include regulation of nervous system development, tissue morphogenesis, head development, and embryonic morphogenesis (Figure 5B). These results suggest a potential dual regulatory role for FOXH1 in promoting pluripotency genes and inhibiting the inappropriate activation of differentiation genes.

To further elucidate the mechanisms through which the transcription factor FOXH1 sustains pluripotency and suppresses differentiation, we performed chromatin immunoprecipitation sequencing (ChIP‐seq) in undifferentiated pEPSCs. A total of 9045 common FOXH1 binding sites were identified in two biological replicates (Figure 5C; Table S5, Supporting Information). Further analysis of the distribution of these genomic binding sites revealed that the majority (76.52%) are located in promoter regions, with fewer in gene bodies (13.15%) and intergenic regions (10.33%). Additionally, these sites are notably enriched within ±1 kb of transcription start sites (TSS), and a substantial overlap of 82.44% with POU5F1 binding sites was observed (Figure 5D). To explore the interaction between FOXH1 and POU5F1, we conducted co‐immunoprecipitation (Co‐IP) assays in pEPSCs. Our initial results indicated that using an anti‐POU5F1 antibody, FOXH1 was effectively co‐precipitated, suggesting a potential interaction between these two transcription factors. Reciprocal Co‐IP experiments further demonstrated that FOXH1 was able to co‐precipitate POU5F1 as well (Figure 5E). These findings highlight the pivotal role of FOXH1 in regulating pluripotency‐associated transcriptional networks through cooperative binding with POU5F1 at key regulatory regions.

*Cis*‐regulatory elements (CREs) serve as critical mediators of pluripotency‐associated gene expression by orchestrating transcriptional activity within their genomic neighborhoods.^[^ ^42^, ^43^ ^]^ For instance, POU5F1 modulates its own expression and regulates pluripotency networks by binding to multiple CREs.^[^ ^44^ ^]^ Recognizing the essential role of CREs in maintaining pluripotency, we employed a multi‐omics strategy to systematically identify potential CREs in pEPSCs (Figure 5F). This included ChIP‐seq profiling of histone modifications (H3K4me3, H3K27ac, and H3K4me1) and ATAC‐seq analysis in WT cells. Through this approach, we characterized active promoters and enhancers, distinguished by specific histone marks and accessibility profiles. Specifically, we delineated 13612 active promoters (defined by H3K4me3 enrichment and chromatin accessibility) and 23036 enhancers (marked by H3K4me1/H3K27ac co‐enrichment and open chromatin states). To further investigate the regulatory role of FOXH1 in maintaining cellular pluripotency, we integrated RNA‐seq data from both differentiation experiments^[^ ^35^ ^]^ and *FOXH1* knockout studies. This analysis identified 45 genes potentially targeted by FOXH1 that are associated with pluripotency (Figure 5G). Subsequent FOXH1 ChIP‐seq analyses confirmed its direct binding within the regulatory regions of these genes (Figure 5H). Notably, ATAC‐seq profiling in FOXH1‐depleted cells revealed significant reductions in chromatin accessibility at these CREs (Figure 5I). These findings collectively suggest that FOXH1 mediates transcriptional regulation of pluripotency‐associated target genes by modulating the chromatin structure of their CREs.

Among the top 10 pluripotency gene related CREs exhibiting the most substantial changes following the deletion of *FOXH1*, the list included not only established pluripotency regulators such as *FZD5*, *ESRRB*, *LEFTY2* and *NODAL* but also *FOXA2* (Figure S6A, Supporting Information; Figure 5J). Given the swift reduction in FOXA2 mRNA and protein levels following *FOXH1* KO (Figure 4C,D), coupled with the ambiguous role of FOXA2 in pluripotency, we propose that FOXA2 is a potential FOXH1 target involved in pluripotency maintenance. To validate this hypothesis, we ectopically overexpressed *FOXA2* in *FOXH1* KO cells (Figure S6B, Supporting Information). We observed that overexpression of *FOXA2* rescued the flattened cell phenotype and disrupted cell junctions induced by *FOXH1* loss (Figure 5K). Furthermore, RT‐qPCR showed that *FOXA2* overexpression partially restored the expression of the pluripotency marker *NANOG*, *FOS*, and the three germ layer markers *EOMES*, *KRT8*, and *CDH1* (Figure 5L; Table S4, Supporting Information). This outcome implies that while *FOXA2* overexpression cannot fully substitute for the function of FOXH1, it suggests a potential upstream regulatory role of FOXH1 over FOXA2.

To further verify the direct regulatory linkage between FOXH1 and FOXA2, we engineered a reporter vector encompassing ≈3000 bp of the *FOXA2* promoter sequence and co‐transfected this construct with a *FOXH1* overexpression vector into HEK293T cells. We observed that *FOXH1* overexpression significantly increased the transcription and fluorescence intensity of mCherry (Figure S6C and Table S4, Supporting Information), indicating a direct regulatory effect of FOXH1 on FOXA2. Consequently, we deduce that the direct modulation of FOXA2 by FOXH1 also persists during the maintenance of pluripotency, likely through the regulation of chromatin accessibility at CREs to promote FOXA2 expression.

In summary, our results underscore the vital role of FOXH1 in regulating its target genes related to pluripotency by sustaining their open chromatin configuration.

### 2.5 FOXH1 Loss Increased H3K4me3 Deposition at Promoters of Target Differentiation‐Related Genes

**Figure 6:**
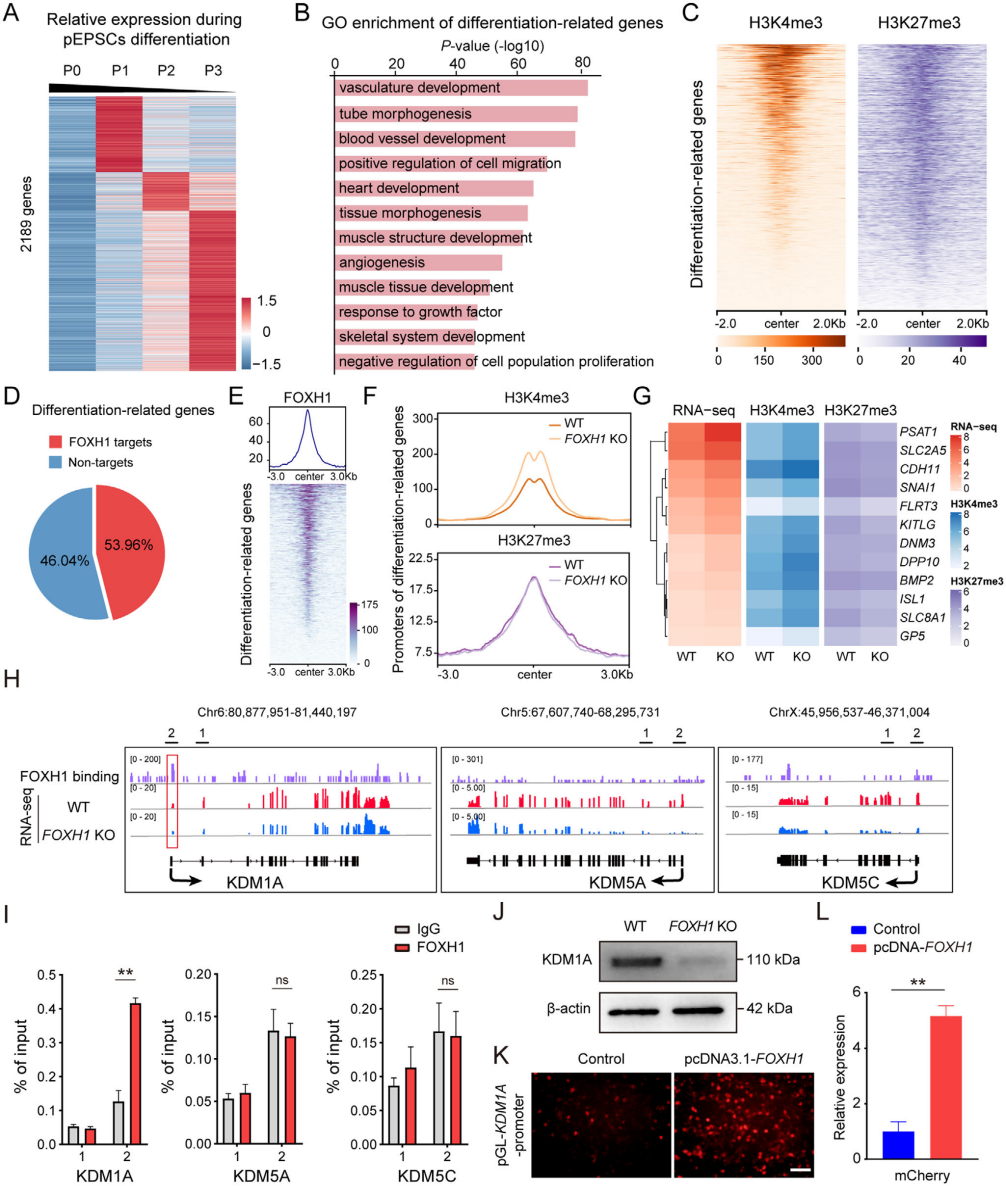
FOXH1 modulates lineage‐specific genes expression through regulation of H3K4me3 during pluripotency maintaining. A) Heatmap displaying the relative expression of 2189 genes during the differentiation of pEPSCs based on published RNA‐seq data. B) GO enrichment analysis of differentiation‐related genes. C) Heatmaps depicting the distribution of H3K4me3 and H3K27me3 marks across differentiation‐related genes. D) Pie chart representation of FOXH1 target genes versus non‐targets within the differentiation‐related gene set. E) Distribution of FOXH1 binding intensity across differentiation‐related gene promoters shown as a density plot and heatmap. F) Comparative analysis of H3K4me3 and H3K27me3 levels at differentiation‐related gene promoters in WT and *FOXH1* KO cells. G) Multi‐panel display of RNA‐seq and histone modification profiles (H3K4me3, H3K27me3) for selected lineage‐specific genes in WT and *FOXH1* KO cells. H) The IGV snapshots show RNA‐seq of the promoter regions of *KDM1A*, *KDM5A*, and *KDM5C* in WT and *FOXH1* KO cells, highlighting FOXH1 binding specifically in the promoter region of *KDM1A*. The region labeled as “2′ represents the proximal promoter region, while the region labeled as “1′ serves as a negative control for ChIP‐qPCR verification. I) ChIP–qPCR analysis of the enrichment of FOXH1 at the KDM1A, KDM5A, and KDM5C loci. J) Western blot analysis of KDM1A expression in WT and FOXH1 KO cells. K) Representative images of HEK293T cells co‐transfected with empty vector or FOXH1 overexpression vector and KDM1A‐promoter‐mCherry reporter vector. Scale bars, 100 µm. L) Quantification of mCherry expression. Data are shown as mean ± SD. ^**^ *p* < 0.01; two‐tailed Student's t test.

Considering the RNA‐seq data from the *FOXH1* KO showed upregulation of genes critical for differentiation, we next interested in exploring how FOXH1 regulates genes involved in lineage differentiation. By analyzing our previously published RNA‐seq datasets,^[^ ^35^ ^]^ we identified 2189 genes upregulated during pEPSC differentiation, subsequently classified as differentiation‐related genes (**Figure** **6**A). GO analysis revealed that these genes were strongly associated with differential GO terms, including vasculature development, tube morphogenesis, blood vessel development, tissue morphogenesis, and others (Figure 6B). Given that the histone modifications H3K4me3 and H3K27me3 predominantly mark developmental and lineage‐specific genes that are silenced yet easily activated,^[^ ^45^, ^46^ ^]^ further analysis via ChIP‐seq for H3K4me3 and H3K27me3 confirmed that a majority of the identified differentiation‐related genes possess these modifications (Figure 6C). Importantly, FOXH1 was found to bind significantly to the promoters of these genes, many of which are direct FOXH1 targets (Figure 6D,E). This finding suggests that FOXH1 may play a crucial role in sustaining the undifferentiated state of pEPSCs by directly modulating these differentiation‐related genes.

Subsequently, we investigated whether FOXH1 modulates transcription levels of its target differentiation‐related genes by regulating key histone modifications. Our analysis revealed that in the absence of FOXH1, there is a notable increase in the levels of H3K4me3 at the promoters of differentiation‐related genes, without significant changes in the levels of H3K27me3 (Figure 6F). Furthermore, the ablation of FOXH1 resulted in enhanced H3K4me3 binding at the promoters of genes such as *CDH11*, *DPP10*, *BMP2*, and *ISL1*, while H3K27me3 levels remained stable (Figure 6G). Taken together, these changes activated the transcription of these genes. This suggests that FOXH1 maintains pluripotency in pEPSCs by orchestrating the integrity of the bivalent domain.

Previous investigations have demonstrated that histone demethylases are implicated in the suppression of gene expression by removing the H3K4me3 modification from promoter regions.^[^ ^47^, ^48^ ^]^ Considering the enhanced H3K4me3 signals following *FOXH1* deletion, we further investigated whether the expression levels of genes encoding H3K4me3‐associated demethylases were impacted. Comparative RNA‐seq and RT‐qPCR analysis between WT and *FOXH1* KO pEPSCs revealed a notable downregulation of *KDM1A*, along with a reduction in *KDM5C* and *KDM5A* expression (Figure S6D and Table S4, Supporting Information). These enzymes, which remove methyl groups from H3K4, likely contribute to the rise in H3K4me3 levels due to their decreased expression.^[^ ^49^, ^50^ ^]^ Consequently, we hypothesized that FOXH1 might influence the expression of these demethylase genes either directly or indirectly, thereby affecting H3K4me3 levels and regulating the expression of differentiation‐associated genes. To elucidate the potential regulatory roles of FOXH1 on these demethylases, we integrated both ChIP‐seq and RNA‐seq datasets to assess whether FOXH1 binds directly to the promoter regions of these genes to regulate their transcription. Notably, Integrative Genomics Viewer (IGV) analysis revealed FOXH1 occupancy at the *KDM1A* promoter region (Figure 6H). ChIP‐qPCR further confirmed the specific binding of FOXH1 to the *KDM1A* promoter (Figure 6I). Consistently, the protein expression of KDM1A was significantly reduced in *FOXH1* KO cells (Figure 6J). However, no significant binding was detected at the promoters of *KDM5C* and *KDM5A* (Figure 6H,I). These results suggest that FOXH1 may directly regulate the expression of KDM1A and indirectly influence the expression of KDM5C and KDM5A, thereby modulating the enrichment levels of H3K4me3. It has been reported that inhibition of KDM1A activity in ESCs impedes their differentiation and prevents the downregulation of pluripotency‐associated genes.^[^ ^51^ ^]^ To further validate the regulatory role of FOXH1 on KDM1A, we constructed a mCherry reporter vector that contains ≈3000 bp of the *KDM1A* promoter sequence. *FOXH1* overexpression markedly increased both the transcription and fluorescence intensity of mCherry (Figure 6K,L; Table S4, Supporting Information), demonstrating a direct regulatory effect of FOXH1 on KDM1A. Collectively, these findings illustrate that FOXH1 directly controls KDM1A to adjust H3K4me3 levels at the promoters of target differentiation‐related genes, thus maintaining the pluripotency of pEPSCs.

## 3 Discussion

pEPSCs have great potential for applications in regenerative medicine and stem cell‐based breeding programs. However, the regulatory mechanisms governing pluripotency in pEPSCs, including aspects of cellular adaptability and the maintenance of a pluripotent identity, are not yet fully understood. Moreover, challenges such as low rates of chimeric integration and limited germ line competency hinder their broader application.^[^ ^5^ ^]^ Therefore, exploring whether pluripotency is regulated by mechanisms similar to those controlling cellular adaptability will enhance our understanding of pluripotency in pEPSCs and expand their practical utility.

In recent years, numerous transcription factors and epigenetic regulators have been demonstrated to play critical roles in maintaining pluripotency.^[^ ^52^, ^53^, ^54^, ^55^ ^]^ However, the functional roles of many related genes remain insufficiently uncovered. To investigate their roles in preserving the essential characteristics of pEPSCs, we conducted two types of CRISPR‐Cas9 knockout screening of all annotated TFs and epigenetic regulators in pigs. The first screening focused on identifying genes essential for cell viability and proliferation, termed fitness screening. We identified 900 essential genes, including *POU5F1* and *MYC*, known to be highly relevant to ESC adaptability,^[^ ^30^, ^31^ ^]^ thereby validating the effectiveness of our screening method. Additionally, by integrating data from screenings conducted across various species,^[^ ^22^, ^32^, ^33^, ^34^ ^]^ we identified conserved genes across species, such as *PRMT1*, *MYBL2*, and *PCNA*, as well as porcine‐specific genes, *NASP* and *FOS*, which are crucial for maintaining the fundamental biological activities of pEPSCs. Interestingly, the essential genes in human or mouse, such as *ELP4*, *SALL4*, *ZPR1* and *CHD8*, were not deemed essential in pigs,^[^ ^22^, ^32^ ^]^ underscoring the unique role of certain genes within species‐specific contexts. In the subsequent screenings focused on pluripotency, by utilizing FACS sorting based on POU5F1 and NANOG protein expression, we identified genes that contribute to the pluripotency identity rather than to cellular fitness, as these were not listed among the essential genes. This suggests that studying pluripotency solely from the perspective of stem cell fitness is insufficient. Our screening revealed several known pluripotency regulators such as *ZFP42*, *SOX2*, and *OTX2*, as well as novel candidate genes including *FOXH1*, *SRSF2*, *CSNK2A1*, and *TRIM24*, which warrant further investigation in pEPSCs. Based on its distinctive knockout phenotype, FOXH1 was selected for more further investigation. Here, our study shows for the first time that FOXH1 is a pivotal factor in maintaining pluripotency in pEPSCs. FOXH1 not only sustains the expression of pluripotency genes but also plays a crucial role in inhibiting the premature expression of differentiation‐related genes. Overall, our research establishes a novel screening method and provides additional candidate genes, laying the foundation for deeper exploration into the mechanisms controlling cellular pluripotency.

FOXH1 is expressed during the early embryonic stages and acts as a master regulator of mesendoderm specification.^[^ ^56^ ^]^ Embryos lacking FOXH1 exhibit defects in mesendoderm development in zebrafish and *Xenopus laevis*, as well as in mouse.^[^ ^57^, ^58^, ^59^ ^]^ Although previous research has demonstrated FOXH1 expression in pluripotent stem cells, *FOXH1* depletion by siRNA or CRISPR‐Cas9 does not impact the pluripotency maintenance in human pluripotent stem cells (hPSCs).^[^ ^32^, ^41^, ^60^ ^]^ However, our findings indicate that FOXH1 is crucial for maintaining pluripotency in pEPSCs. Loss of FOXH1 under pluripotency culture conditions induces significant alterations in cell morphology, reduces the expression of pluripotency markers, and shifts gene expression patterns toward stem cell differentiation. Additionally, *FOXH1* knockdown significantly impedes the formation of porcine blastocysts, highlighting its role at an earlier stage of porcine embryonic development. Furthermore, during reprogramming of human somatic cells induced by POU5F1, SOX2, KLF4, and MYC (OSKM), which transiently induces a mesendoderm‐like state, FOXH1 significantly improves efficiency by facilitating the maturation of intermediate cells.^[^ ^61^, ^62^ ^]^ Surprisingly, our observations reveal that overexpressing *FOXH1*, based on OSKM, actually reduces reprogramming efficiency of pig somatic cells and is accompanied by massive cell death (unpublished data). These findings underscore the complex role of FOXH1 across different species and cell types, offering new insights into its potential as a key factor within the pluripotency regulatory network of porcine pluripotent cells.

To elucidate how FOXH1 contributes to the regulatory landscape of pluripotency in pEPSCs, we conducted a comprehensive analysis by integrating multi‐omics data. Our findings suggest a dual mechanism by which FOXH1 governs these processes (Figure S6E, Supporting Information). First, it is well‐established that chromatin accessibility is closely linked to transcriptional activity, and cellular state transitions are often accompanied by chromatin remodeling.^[^ ^63^ ^]^ FOXH1 is demonstrated to sustain chromatin accessibility by facilitating local chromatin decondensation.^[^ ^64^, ^65^ ^]^ Consistent with this notion, in our study, we found that FOXH1 maintains an open chromatin configuration at the promoter regions of its target pluripotency genes, thus facilitating their transcriptional activity. Secondly, FOXH1 modulates differentiation pathways by regulating critical histone modifications, such as H3K4me3, which is generally associated with active transcription.^[^ ^66^, ^67^ ^]^ FOXH1 maintains a balance of H3K4me3 and H3K27me3 modifications around the promoters of targeted differentiation genes. This balance prevents the premature or inappropriate activation of these genes during the maintenance of pluripotency. Further investigations are essential to determine whether FOXH1 interacts with other transcription factors to sustain chromatin accessibility and whether it recruits additional epigenetic modification complexes to regulate gene expression. Anticipated future research is expected to uncover more refined regulatory mechanisms and deepen our understanding of the cellular pluripotency maintenance processes.

**Figure 7:**
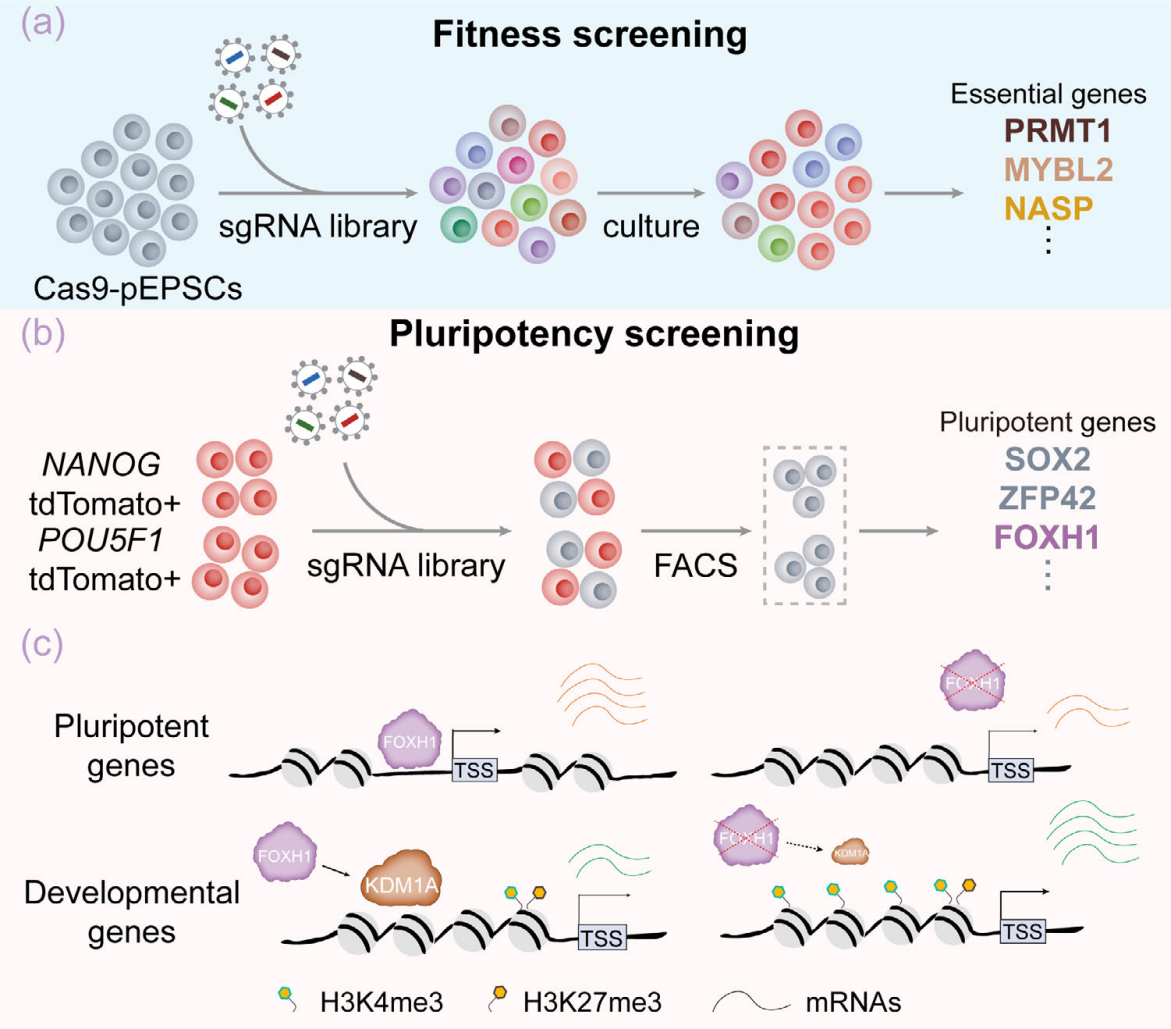
Schematic illustration of the major findings of this study.

In conclusion, through functional genomic screenings, we have successfully identified key genes that are essential for the self‐renewal and maintenance of pluripotency in pEPSCs. Our study highlights the pivotal role of FOXH1 as a core regulatory factor in maintaining pEPSCs pluripotency, emphasizing the critical importance of conducting species‐specific research (**Figure** **7**). It would be meaningful to apply the existing technologies and analytical methods used in this study to other culture‐dependent pESCs or across various genetic backgrounds. The resources developed through this study not only lay the groundwork for more extensive CRISPR screenings but also provide crucial theoretical support for the establishment of pPSC cell lines with higher differentiation potential.

## 4 Experimental Section

### Animal Ethics Statements

All animal experimental procedures were approved in advance by the Animal Care and Use Committee of Huazhong Agricultural University (HZAUMO‐2024‐0034).

Porcine EPSC cells were derived as previously reported.^[^ ^5^ ^]^ Briefly, porcine ICMs were isolated by mechanical isolation and treated with TrypLE Express (Thermo Fisher Scientific, 12604‐013) for 3 min, then seeded onto mitomycin C‐treated STO feeder cells supplemented with pEPSC medium (pEPSCM) and 10 µM Y27632 (Tocris, 1254/1).^[^ ^5^ ^]^ Outgrowths observed within 10 days were dissociated with 0.05% Trypsin/EDTA (Thermo Fisher Scientific, 25 300 062) for 3 min and passaged onto feeder cells in pEPSCM with 5 µm Y27632 and 5% fetal bovine serum (FBS). After 12–24 h, the medium was replaced with fresh pEPSCM, and then passaged every 3–4 days at a ratio of 1:3. pEPSCM (50 mL) was prepared as follows: 48.25 mL KO‐DMEM (Thermo Fisher Scientific, 10829‐018), 0.25 mL N2 supplement (Thermo Fisher Scientific, 17502‐048), 0.5 mL B27 supplement (Thermo Fisher Scientific, 17 504 044), 0.5 mL Gluta MAX (Thermo Fisher Scientific, 35050–061), 0.5 mL NEAA (Thermo Fisher Scientific, 11140‐050), 0.5 mL Penicillin‐Streptomycin (Thermo Fisher Scientific, 15140‐122), 0.1 mm 2‐mercaptoethanol (Sigma, 21985‐023), 0.2 µm CHIR99021 (GSK3i; Tocris, 4423), 0.3 µm WH‐4‐023 (Tocris, 5413), 2.5 µm XAV939 (Sigma, X3004), 65.0 µg mL^−1^ vitamin C (Sigma, A5960), 10.0 ng mL^−1^ Recombinant Human LIF (Peprotech, 300–05), 20.0 ng mL^−1^ Activin A (Peprotech, 120‐14P) and 0.3% FBS (Hyclone, SH20070.03). HEK293T cells were cultured in DMEM medium (Gibco, C11965500BT) supplemented with 10% FBS (Vistech, SE200‐ES), 1% GlutaMAX (Gibco, 35 050 061), and 1% Penicillin‐Streptomycin (Gibco, 15 140 122). All Cells were cultured at 37 °C, 20% O_2_, and 5% CO_2_.

### Alkaline Phosphatase (AP) Staining

The pEPSCs were fixed in 4% paraformaldehyde (PFA) in PBS for 10 min at room temperature and then washed three times with ice‐cold PBS. After that, the cells were incubated with a mixture of 1.0 mg/mL Fast Red TR (Sigma, F6760) and 0.4 mg/mL Naphthol AS‐MX (Sigma, N4875) in 0.1 m Tris buffer (pH 8.2) at room temperature for 30 min.

### Karyotype analyses

Before performing karyotype analyses, pEPSCs were treated with 1% KaryoMAX Colcemid Solution (Thermo Fisher Scientific, 15 212 012) in a fresh culture medium for 2 h. The cells were dissociated into single cells, centrifuged, resuspended in 0.075 m KCl (Sigma, P5405) hypotonic solution, and incubated at 37 °C for 15 min. Following this step, pEPSCs were fixed with methanol and acetic acid in a ratio of 3:1, and this step was repeated three times. The resulting suspension of pEPSCs was dropped onto precooled slides and stained with a 10% Giemsa Stain Solution (Beyotime, C0133).

### Immunofluorescence Staining

pEPSCs were fixed with 4% PFA for 30 min at room temperature, washed in PBS for 5 min, and permeabilized with 0.5% Triton X‐100 in PBS for 45 min. Then, the cells were blocked with 0.1% bovine serum albumin (BSA) for 1 h at room temperature. The pEPSCs were then incubated with primary antibody (POU5F1, Abcam, ab181557, 1:200; NANOG, peprotech, 500‐p236, 1:100; SOX2, SantaCruz, sc‐365823, 1:200; FOXA2, Abcam, ab108422, 1:200; FOXH1, Abcam, ab229241, 1:100; LEF1, Abcam, ab137872, 1:100; EOMES, Abcam, ab216870, 1:100; T, Abcam, ab209665, 1:250; KRT8, Abcam, ab53280, 1:100; SOX1, CST, 41 945, 1:100; FGF5, Abcam, ab88118, 1:100; S0×17, R&D, AF1924, 1:200; GATA6, CST, 5851, 1:100) overnight at 4 °C. After washed in PBS three times, the cells were incubated with fluorescent‐dye conjugated secondary antibodies for 1 h at room temperature. Blastocysts were fixed with 4% PFA for 30 min and then washed three times with PBS containing 0.05% polyvinyl alcohol. Blastocysts were then permeabilized with 0.5% Triton X‐100 for 30 min, blocked with 5% BSA for 2 h, and incubated at 4 °C overnight with primary antibodies (KLF4, Abcam, ab215036, 1:200; CDX2, Biogenex, MU392A‐UC, 1:100). The blastocysts were incubated with fluorescent‐dye conjugated secondary antibodies for 1 h at room temperature. Finally, samples were counterstained with DAPI (Roche Life Science, 10 236 276 001) for 5 min at room temperature. Stained cells or blastocysts on glass slides were observed using a Zeiss confocal microscope (Zeiss LSM 800).

### Western Blot

Western blotting was performed as described previously.^[^ ^68^ ^]^ Antibodies used were anti‐Cas9 (1:1000, Abclonal, A14997), POU5F1 (1:1000, Abcam, ab181557), NANOG (1:500, peprotech, 500‐p236), FOXA2 (1:1000, Abcam, ab108422) and β‐actin (1:1000, proteintech, 66 009).

### Porcine CRISPR sgRNA Library Design

The porcine transcription factors and epigenetic‐related factors were selected based on previously published literature^[^ ^24^ ^]^ and the AnimalTFDB 3.0 database (http://bioinfo.life.hust.edu.cn/AnimalTFDB/#!/tf_summary?species=Sus_scrofa). Four sgRNAs per gene were designed using the CRISPR offinder software^[^ ^69^ ^]^ (version 1.2, http://www.biootools.com). Sequences of all porcine transcription factors and epigenetic‐related factors were obtained from the Ensembl database (version 10.2, www.ensembl.org/index.html). The porcine CRISPR knockout library contains 11440 sgRNAs, including 10440 sgRNAs targeting 2610 protein‐coding genes and 1000 negative control sgRNAs that do not target any genomic sequences. The selected sgRNAs were weighted based on targeting the first 50% of the open reading frames and minimizing potential off‐target sites. The selection criteria for the sgRNAs allowed for a maximum of three nucleotide mismatches within the 20‐mer targeting region, to avoid overlap between sgRNAs targeting the same genomic loci. Details regarding the features of the porcine CRISPR knockout library are provided in Table S2 (Supporting Information).

### Construction of a Porcine CRISPR sgRNA Library Plasmid

The sgRNA library was constructed as previously reported.^[^ ^24^, ^35^ ^]^ Briefly, the library was synthesized using CustomArray 90 K arrays (CustomArray Inc.) and amplified by PCR using Phusion High‐Fidelity PCR Master Mix with HF Buffer (NEB) to produce sub‐pools for Gibson assembly (NEB). The PCR reaction was performed in a Veriti 96‐Well Thermal Cycler (Thermo Fisher Scientific) for 16 cycles. In total, 40 PCR reactions were performed using 50 ng of oligo pool per 50 µL of reaction volume. The PCR products were mixed and purified using a MinElute PCR Purification Kit (QIAGEN) and then ligated into the linearized lenti‐sgRNA‐puro‐ZsGreen vector using Gibson assembly. The ligation mixtures were transformed into Trans1‐T1 Phage Resistant Chemically Competent Cells (Transgen). To achieve sufficient coverage, parallel transformations were performed, counting the number of colonies to reach 200 times the total sgRNAs in the library. The sgRNA library plasmids were extracted with a Plasmid Plus Maxi Kit (QIAGEN). The library plasmids were amplified using PrimeSTAR GXL DNA Polymerase (Takara) for 16 reaction cycles. The PCR products were purified using a QIAquick Gel Extraction Kit (QIAGEN) and then analyzed by high‐throughput sequencing to examine the sgRNA coverage in the library plasmids. All primers for constructing the sgRNA expression vector are listed in Table S4 (Supporting Information).

### Porcine CRISPR sgRNA Library Lentivirus Production and Transduction

Lentiviral particles were generated by co‐transfecting HEK293T cells with a mixture of plasmids: 9 µg of the sgRNA library plasmid, 6 µg of the psPAX2 packaging plasmid, and 3 µg of the pMD2.G envelope plasmid (both from Addgene) per 100 mm dish. Transfections were performed using JetPRIME (PolyPlus) following the manufacturer's protocol to ensure optimal transfection efficiency. Viral supernatants were harvested 48 h and 72 h post‐transfection, collected into 50 mL centrifuge tubes, and subjected to centrifugation at 1,500 rpm for 10 min. The clarified supernatant was then filtered through a 0.45 µm filter. To this filtered supernatant, PEG 8000 was added as a lentiviral concentrate, mixed thoroughly, and incubated at 4 °C overnight to facilitate viral precipitation. The precipitated viral particles were resuspended in pEPSCM, dispensed into smaller volumes, and subsequently stored at −80 °C for long‐term preservation. At 24 h post‐transduction, the medium containing the viruses with 4 µg mL^−1^ polybrene (sigma) were discarded and replaced with a fresh medium to continue the cell culture under optimal conditions.

### Generation of Mutant Cell Libraries

A total of 2 × 10^8^ cas9‐expressing cells or NANOG/POU5F1‐tdTomato cells were seeded onto Matrigel (Corning, 354 277), in order to minimize interference from feeder cells, and infected with the library lentiviruses at an MOI of less than 0.3. 24 h after infection, the medium was replaced with fresh medium. Another 24 h later, ≈4 × 10^7^ cells from total cells were extracted using a Blood & Cell Culture DNA Midi Kit (QIAGEN) and expanded for the deep sequencing analysis to examine the coverage of the initial mutant cell libraries.

### Negative and Positive Screening of Mutant Cell Libraries

For the negative CRISPR screening, ≈1 × 10^8^ Cas9‐expressing mutant cells were seeded onto feeder cells and cultured up to 30 days after puromycin selection. At multiple time points post‐infection, viable cells were collected and expanded for the deep sequencing analysis. For the positive CRISPR screening, ≈1 × 10^8^ NANOG/POU5F1‐tdTomato mutant cells were seeded onto feeder cells and cultured for 3–4 generations after puromycin selection. Then, cells were collected and subjected to flow sorting (Beckman) to isolate those that negative or show diminished expression of tdTomato. The selected cells were then collected, expanded, and prepared for deep sequencing analysis to further investigate the effects of the knockout on pluripotency.

### Illumina Sequencing of sgRNAs in the CRISPR Library and Enriched Mutants

Genomic DNA was extracted from each sample using a Blood & Cell Culture DNA Midi Kit (QIAGEN). The sgRNA‐encoding regions were amplified by PCR using Q5 Hot Start High‐Fidelity DNA Polymerase (NEB) in a reaction volume of 50 µL. The PCR products were mixed and purified using the MinElute PCR purification Kit (QIAGEN). The purified PCR products were amplified by PCR using different barcoded primers. All PCR products were pooled and purified with a MinElute PCR purification Kit (QIAGEN), followed by Illumina HiSeq 6000 Next‐generation sequencing. Mapped read counts were subsequently used as input for the MAGeCK (version 0.5) analysis software package.

### Knockout of Candidate Genes in pEPSCs by CRISPR/Cas9

Individual sgRNA targeting to candidate gene was cloned into the linearized lenti‐sgRNA‐puro‐ZsGreen and lentivirus were produced as described above. The resulting lentivirus was transduced into *NANOG*/*POU5F1*‐tdTomato cells or Cas9‐expressing cells. Cells with ZsGreen expression were manually picked and then seeded into 96‐well plates to generate clonal knockout. At the seventh day after transduction, the genotypes of cell colonies were analyzed by extracting genomic DNA (TIANamp Genomic DNA Kit, TIANGEN) and sequencing. All primers for identifying the genotype of cell colonies are listed in Supplemental Table S4 (Supporting Information).

### T7 Endonuclease I Cleavage Detection Assay

Potential off‐target sites with high homology in the sgRNAs were predicted using CRISPR‐offinder. Genomic DNA was extracted from mutated clonal cells using the TIANamp Genomic DNA Kit (TIANGEN) for PCR amplification, and the T7 endonuclease I (T7EN I) assay was conducted to assess off‐target cleavage. Off‐target and target site lesions induced by CRISPR/Cas9 were quantified by PCR amplification of the regions using LA Taq (TaKaRa), followed by T7EN I digestion and analysis on 1.5% agarose gels stained with GelRed. Densitometry of the gels was performed using Image Lab software (Bio‐Rad) to evaluate the insertions and deletions (indels) resulting from non‐homologous end joining (NHEJ).

### In Vitro Transcription, Transfection and Microinjection

Target‐specific interference sequences for porcine *NANOG* and *FOXH1* were designed using the DSIR siRNA design platform. The *POU5F1* interference sequences were derived from previous studies.^[^ ^68^ ^]^ The sense strands of the synthetic oligonucleotide duplexes were:

siRNA‐nontargeting control (NC): UUCUCCGAACGUGUCACGUTT;

siRNA‐*POU5F1*: GCUUCCGACUUCGCCUUCUTT;

siRNA‐*NANOG*: CCAGCGAAUCUUCACCAAUTT;

siRNA‐*FOXH1*: CCUUCUUCAGGGACGAUUACG.

Target‐specific DNA oligonucleotides were annealed and subsequently synthesized into siRNA using the T7 RNAi Transcription Kit (Vazyme, TR102). For cell transfection, siRNAs were diluted to 40 µm by sterile water, and 2–4 × 10^5^ pEPSCs were transfected with 5 µL siRNA through the Nucleofector 2b Manual according to the user manual. For embryo microinjection, siRNAs were diluted to 25 µm by sterile water. Approximately 10 pL of siRNAs were microinjected into zygotes using a PiezoXpert micromanipulator (Eppendorf), followed by culture in porcine zygote medium 3 (PZM3) medium at 37 °C in a 5% CO_2_ environment.^[^ ^70^, ^71^ ^]^ PZM3 stock (100 mL) was prepared as follows: 108 mM NaCl (Sigma, S5886), 10 mm KCl (Sigma, P9541), 0.35 mM KH_2_PO_4_ (Sigma, P0662), 0.40 mm MgSO_4_·7H_2_O (Sigma, M1880), 25.07 mm NaHCO_3_ (Sigma, S5761) and 10 µg mL^−1^ Gentamicin (Sigma, G1397). After filtering the PZM3 stock with a 0.22 µm filter, PZM3(10 mL) was prepared as follows: 9.8 mL PZM3 stock, 50 µL Na pyruvate (Sigma, P2256), 20 µL 500 × Gentamicin (Sigma, G1397), 100 µL Glutamine (Sigma, 56‐85‐9), 200 µL Hypotaurine (Sigma, H1384), 100 µL BME Amino Acids Solution (Sigma, B6766), 100 µL MEM Non‐essential Amino Acids (Sigma, M7145), 0.0062 g Ca‐Lactate (Sigma, 867‐56‐1) and 0.03 g BSA (Sigma, A3311).

For mCherry reporter assay, pGL4.25 [FOXA2/KDM1A ‐promoter3K + mCherry + minP] reporter vector and FOXH1 overexpression vectors were co‐transfected into HEK293T cells by JetPRIME (Ployplus, 114‐01) according to the manufacturer's instructions. Fluorescence was observed under an inverted fluorescence microscope (OLYMPUS IX73) at 24 h after transfection, and cells were then harvested for total RNA extraction.

### Colony Formation and Cell Proliferation

For the colony formation assay, pEPSCs were plated at clonal density (50 cells per cm^2^) and cultured for 6 days. AP staining was carried out as described above, and the number of colonies was counted and statistically analyzed. For the proliferation assay, the pEPSCs cells were plated at a density of 10 000 cells per well in 12‐well plates and cultured for 5 days. Viable cells were determined by Trypan blue exclusion and counted with an automated cell counter (Dakewe) daily. Cell counting was not performed on the first day due to the low number of cells.

### Embryoid Bodies Formation

Following digestion into single cells, WT or KO pEPSCs were seeded at a density of 1 × 10^6^ cells per well and cultured for 3–7 days on 35 mm low‐attachment plates in the EB formation medium: Knockout DMEM (Thermo Fisher Scientific, 10829‐018), 15% fetal bovine serum (FBS, Vistech, SE200‐ES), 1% GlutaMAX (Thermo Fisher Scientific, 35050–061), 1% penicillin‐streptomycin (Thermo Fisher Scientific, 15140‐122), 1% NEAA (Thermo Fisher Scientific, 11 140 050), 0.1 mm 2‐mercaptoethanol (Thermo Fisher Scientific, 21985‐023) and 5 µM Y‐27632 (Tocris, 1254/1). Once the embryoid bodies were formed, they were collected into centrifuge tubes, and RNA was extracted to test for the expression of the target gene.

### Real‐Time Reverse Transcription PCR (qRT‐PCR) Analysis

Total RNA was isolated using TRIzol (Invitrogen, 15596–018). RNA was measured using a NanoDrop 2000 spectrophotometer (Thermo Scientific) for assessing RNA quantity and quality. 1 µg total RNA was reverse transcribed to obtain cDNA with the cDNA Synthesis Kit (Vazyme, R212‐01) according to the manufacturer's protocol. For qPCRs, cDNA (1 µL) was used as a template for the 10 µL reaction, and reactions were performed with Universal SYBR qPCR Master Mix (Vazyme, Q511‐02) run on a Bio‐Rad CFX96. Relative expression levels were calculated using the 2^−△△Ct^ method and normalized to *GAPDH*. Statistical significance was assessed using a two‐tailed unpaired Student's t‐test, with a threshold of *p* < 0.05 set for statistical significance. The primers used are listed in Table S4 (Supporting Information).

### Induced Expression of FOXA2

CDs of *FOXA2* was PCR amplified from pEPSCs cDNA, and was inserted into the PiggyBac‐TRE6H‐neoR. Then, the PiggyBac‐TRE6H‐*FOXA2*‐neoR and PBase were transfected into pEPSCs of *FOXH1* KO through the Nucleofector 2b Manual. The positive cells were obtained by G418 screening. *FOXA2* overexpression was induced by doxycycline (1 µg mL^−1^).

ChIP‐seq and ChIP–qPCR: For ChIP‐seq, pEPSCs were crosslinked with 1% formaldehyde for 10 min, and quenched by 125 mm glycine for 5 min rotation at room temperature. Cells were washed three times with cold PBS and a PIC. After centrifugation, cell pellets were lysed in buffer (50 mm HEPES–KOH, 140 mm NaCl, 1 mM EDTA, 10% glycerol, 0.5% NP‐40, 0.25% Triton X‐100, and 50 mm Tris–HCl, pH 8.0) for 10 min at 4 °C. Chromatin was sonicated to 150–300 bp for subsequent experiments. The samples were pre‐incubated with protein A/G Dynabeads (Life Technologies, 10015D), and the beads were then removed. Subsequently, 3 µg of antibody (FLAG, Sigma, F1804; POU5F1, Abcam, ab181557; H3K27me3, Millipore, 07–449; H3K4me3, Abcam, ab8580; H3K4me1, Abcam, ab8895; H3K27ac, Abcam, ab4729) was added, and the sample was rotated overnight at 4 °C. Beads were washed four times with the washing buffer (10 mM Tris–HCl, pH 8.0, 140 mm NaCl, 1 mM EDTA, 1% glycerol, 0.5% Triton X‐100, and 0.01% SDS), eluted, and reverse‐crosslinked. DNA was purified by phenol chloroform and ethanol‐precipitated. Purified DNA was subjected to library construction using KAPA Hyper Prep Kit (KK8500). The products were purified and size‐selected with AMPure XP beads and performed on the Illumina NovaSeq 6000 platform. The DNA was analyzed by qPCR, and the results were presented as the percentage of input, using the specified primers (Table S4, Supporting Information).

### ATAC‐seq

ATAC‐seq was performed as previously reported.^[^ ^72^ ^]^ In brief, ≈50000 cells were washed once with cold PBS and subsequently resuspended in lysis buffer (10 mm Tris‐HCl, pH 7.4, 10 mm NaCl, 3 mm MgCl_2_, and 0.1% IGEPAL CA‐630). The nuclei suspension was then centrifuged at 4 °C before adding the transposition reaction mix from the TruePrep DNA Library Prep Kit V2 for Illumina (Vazyme, TD501). The reaction mix was gently mixed and incubated at 55 °C for 10 min. The ATAC‐seq library was amplified in nine cycles. A Qiagen MinElute Kit was used to isolate DNA. Amplification of ATAC‐seq libraries was subjected to nine cycles. Libraries were purified with a Qiagen PCR Cleanup Kit. The concentration of the library was measured using a KAPA Library Quantification Kit (KK4824) according to the manufacturer's instructions. Gel electrophoresis was used to assess the library's integrity. Lastly, the ATAC libraries were sequenced using the Illumina NovaSeq 6000 platform.

### RNA‐seq and Data Analysis

The WT and FOXH1 KO pEPSCs for RNA‐seq were collected without STO feeder cells and included three biological replicates. Total RNA was extracted by TRIzol and the RNA‐seq library was generated using the RNA Library Prep Kit for Illumina (NEB, E7530L) according to the manufacturer's recommendations. TrimGalore (version 0.6.6) was used to remove adapters and low‐quality reads from RNA‐seq raw reads. These processed reads were then aligned to the pig reference genome using STAR (version 2.7.3) with the default parameters. SAMtools (version 1.9) was used to further analyze mapped reads with high confidence. Stringtie (version 2.1.4) was used to calculate fragments per kilobase per million mapped reads (FPKM) for all Refseq genes, and the FPKM values of replicates were averaged. To perform differential gene expression analysis, FeatureCounts (version 2.0.0) was first used to calculate the read counts of each RNA‐seq sample. Then, in R, differential analysis with Deseq2 was performed. Differentially expressed genes (DEGs) were classified as those with *p* = 0.05 and FC > 1 or FC < −1.

### ChIP‐seq and ATAC‐seq Data Processing

ChIP‐seq and ATAC‐seq raw data were processed by TrimGalore (version 0.6.6) to remove adapters and low‐quality reads using the following parameters “‐q25 –phred33 –stringency 3 –length 36 ‐e 0.1.” The processed reads were then aligned to the pig reference genome (NCBI Sscrofa11.1) using Bowtie2 (version 2.4.4) with parameters “–local –very‐sensitive‐local –no‐mixed –no‐ discordant –phred33 ‐I10 ‐X 700.” PCR duplicates were removed using Picard (version 2.23.9). ChIP‐seq peaks were called with MACS2 (version 2.2.7.1) with the parameters “‐f BAMPE –bdg – SPMR ‐q 0.05 –call‐summits –seed 11 521 –keep‐dup all.” While ATAC‐seq peaks were called with parameters “‐f BAMPE ‐g 2 472 047 704 –shift ‐75 –extsize 150 –keep‐dup all –bdg –cutoff‐ analysis.” Signal tracks for each sample were generated and normalized by calculating the reads per kilobase of transcript per million mapped reads (RPKM) using DeepTools (version 3.5.0). Normalized signals were calculated for promoter regions (defined as [‐1k, 1k] around the TSS) of genes.

### Statistical Analysis

All experiments were repeated at least three times unless otherwise stated. For tdTomato/mCherry reporter assay and immunofluorescence experiments, the average fluorescence intensity per unit area within the area of interest was determined using ImageJ software (version 1.48). Statistical analysis was performed using R (www.r‐project. org/) and GraphPad Prism software. Developmental rates, quantitative PCR results, and fluorescence quantification results were presented as mean ± SD. Two‐tailed Student's t‐test was used to determine significant differences between treatment and control groups (^*^ *P* < 0.05; ^**^ *P* < 0.01; ^***^ *P* < 0.001; ns: no significant). IGV (version 2.11.9) was performed to visualize all sequencing tracks.

## Conflict of Interest

The authors declare no conflict of interest.

## Author Contributions

P.S. and L.H.W. contributed equally to this work. P.S. carried out the experiments. L.H.W. conducted the bioinformatics analyses. D.L.L. and W.T.S. were involved in the identification and knockout of candidate genes. D.G.T. and S.S.X. provided assistance with the design of the porcine CRISPR sgRNA library. L.L. assisted with siRNA microinjection in embryos. T.X., Q.W., and M.X.G. provided support for the cell and molecular experiments. X.L. and X.Z. offered discussion and advice on data analysis. J.L.Z. and Y.‐L.M. supervised the project. P.S., L.H.W., J.L.Z., and Y.‐L.M. wrote the manuscript. All authors have thoroughly reviewed and approved the final manuscript for publication.

## Supporting information



Supporting Information

Supporting Information

Supporting Information

Supporting Information

Supporting Information

Supporting Information

## Data Availability

The raw sequence data and processed counts data that support the findings of this study have been deposited in the National Center for Biotechnology Information's Gene Expression Omnibus and are accessible through GEO Series accession number GSE283822. This paper does not report original code. Any additional information required to reanalyze the data reported in this work paper is available from the lead contact upon request.
