# Supplementary material for: CRISPR Screening Reveals a Novel Role for FOXH1 in Regulating Pluripotency of Porcine Embryonic Stem Cells
Source: Adv Sci (Weinh). 2025 Jul 11;12(34):e09495. doi: 10.1002/advs.202509495 (PMC12442679; doi:10.1002/advs.202509495)
Supplement: Supplementary file 1 — Supporting Information [file ADVS-12-e09495-s004.docx]

**Supplementary Figures**


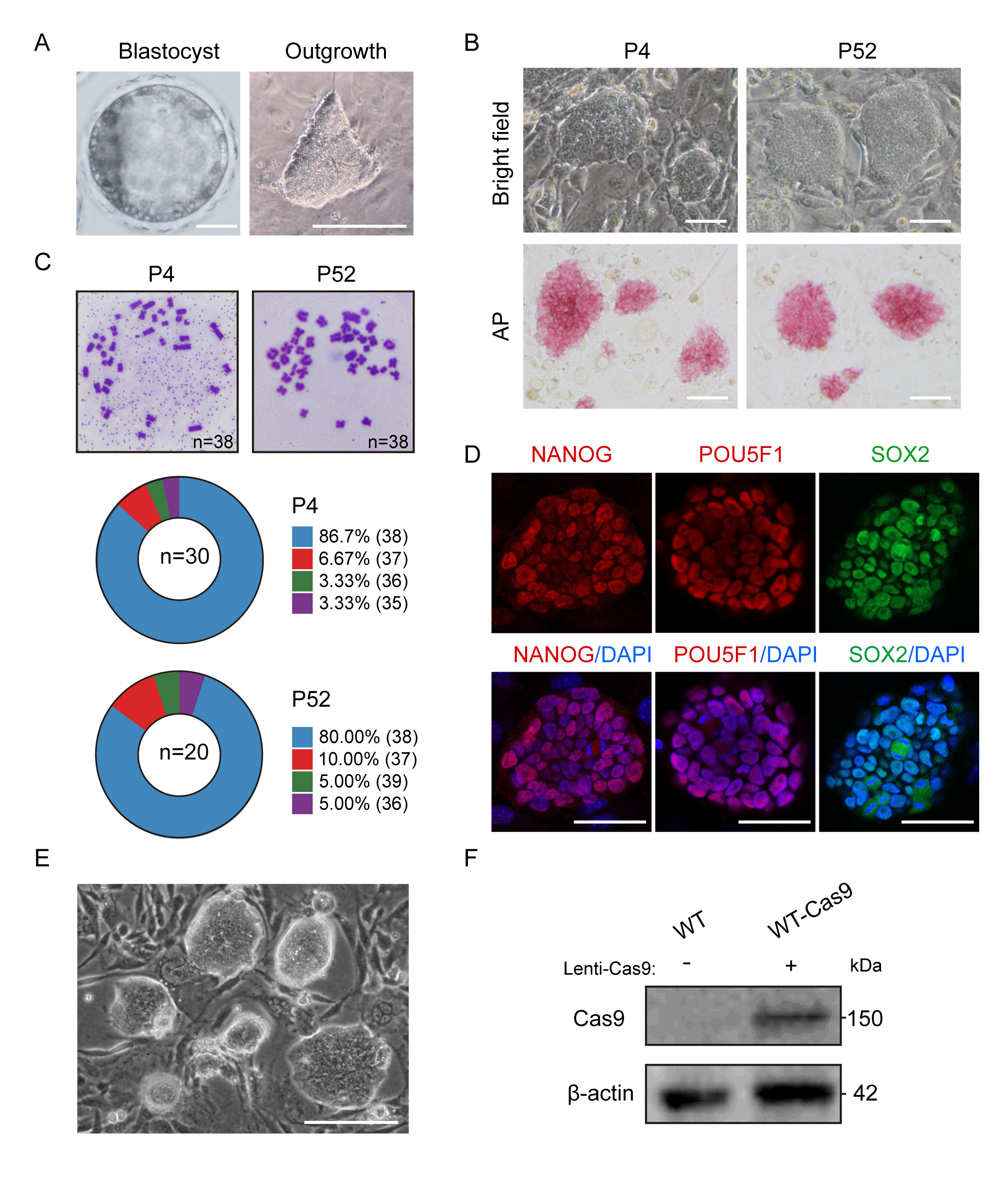


**Figure S1. Derivation of Cas9-expressing pEPSCs.** A) Representative morphology of Bama porcine blastocysts derived *in vivo* and their outgrowths cultured *in vitro*. Scale bar, 50 μm. B) Representative morphology and AP staining of B9 cell line at passages 4 and 52. Scale bar, 50 μm. C) Karyotype analysis of B9 pEPSCs at passages 4 and 52. Bottom panel: Quantification of representative images for chromosome numbers at passages 4 and 52 shows that the majority retain the normal chromosome count of 38. D) Representative immunofluorescent staining for pluripotency markers, including NANOG, POU5F1, SOX2. Scale bar, 50 μm. E) Representative morphology of Cas9-expressing pEPSCs. Scale bar, 100 μm. F) Western blot analysis of Cas9 expression in WT and Cas9-expressing pEPSCs.


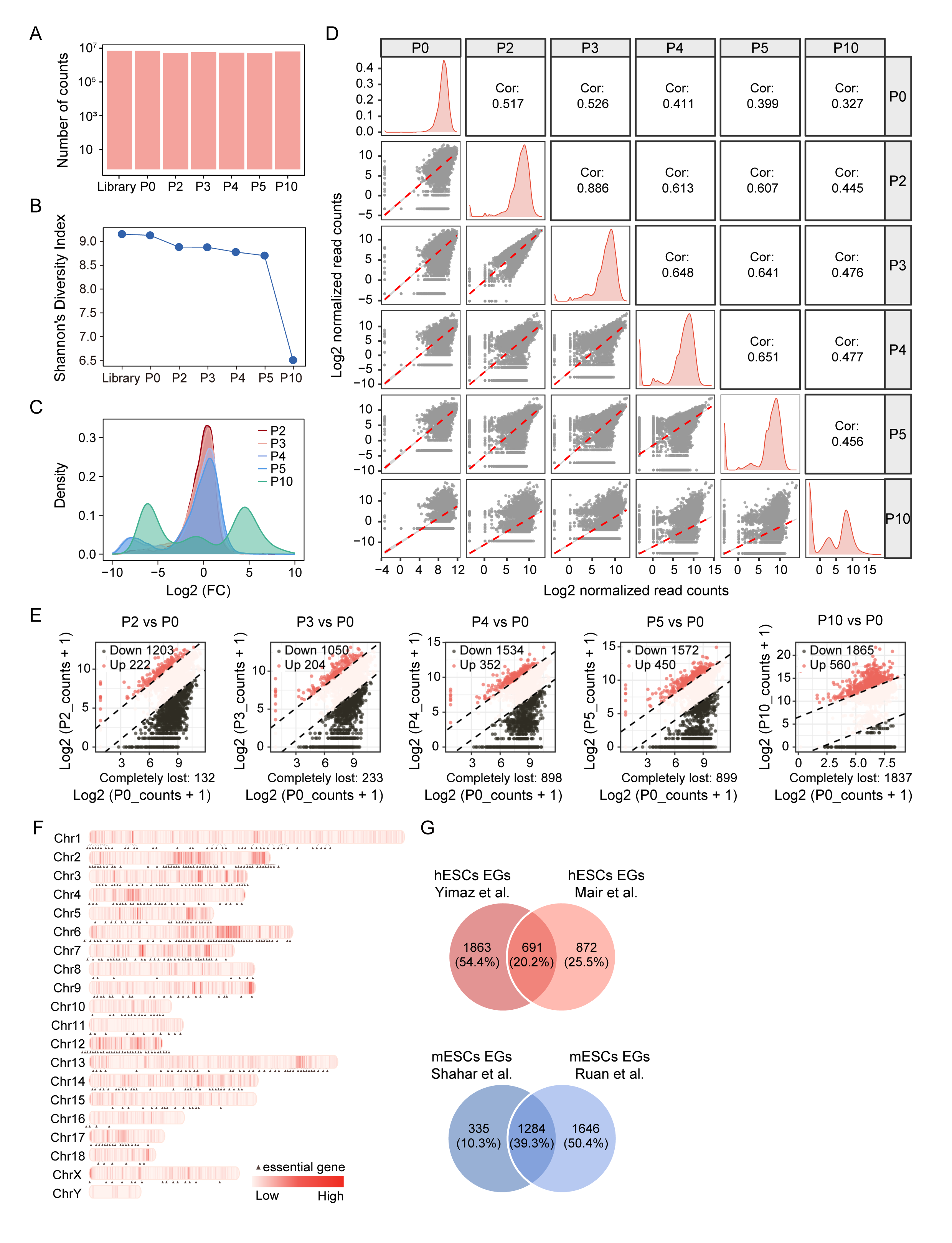


**Figure S2.** **Comprehensive analysis of CRISPR screening across various time points.** A) Histogram showing the sequencing volumes of sgRNAs in the initial plasmid library (Library) and mutation cell libraries at various passages. B) Shannon's Diversity Index for the libraries at various passages, showing a decrease in diversity scores as the proportion of essential-targeting sgRNAs decreases in the population. C) Density plot illustrating the distribution of the Log2 (Fold Change) of gene expression across different passages, with each passage represented by a distinct color. D) Scatter plots with histograms showing the correlation of normalized read counts between various passages (P0, P2, P3, P4, P5, P10). Each panel includes a correlation coefficient (Cor) to indicate the strength of the relationship. E) Changes in gene expression between passages. Each plot compares specific passage differences (P2 vs P0, P3 vs P0, etc.), including genes that are upregulated, downregulated, or completely lost. The number of genes in each category is indicated. F) Chromosomal distribution of essential genes in the library. G) Venn diagrams showing the overlap of essential gene sets in human and mouse ESCs.


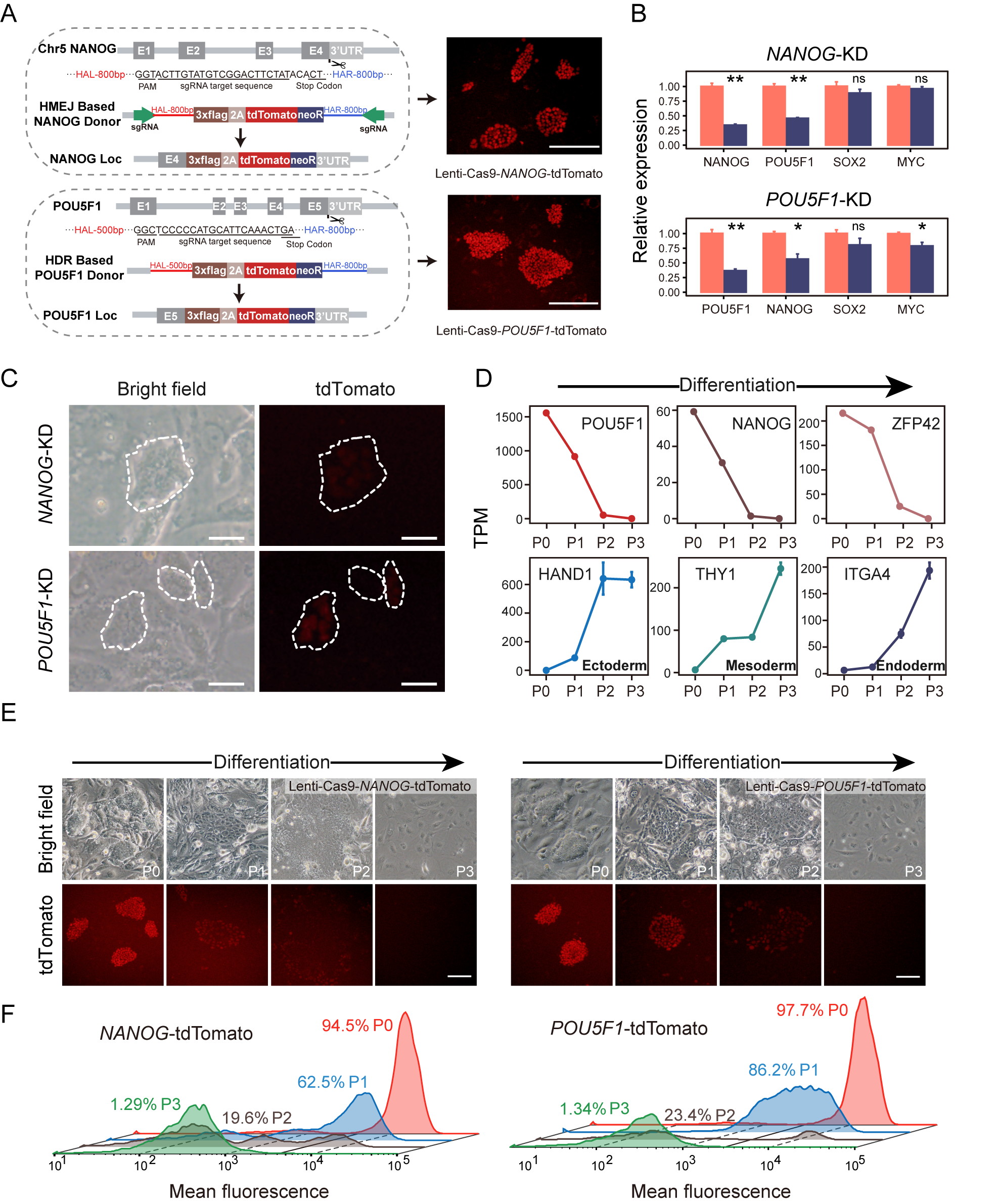


**Figure S3. Establishment and validation of *NANOG*-tdTomato and *POU5F1*-tdTomato reporter cell lines.** A) Scheme of the knock-in strategy (left) and fluorescence microscopy images (right) for the lenti-cas9-NANOG-tdTomato and lenti-cas9-POU5F1-tdTomato cell lines. Scale bar, 100 μm. B) RT-qPCR analysis of expression levels of the pluripotency genes in negative control and *NANOG* KD or *POU5F1* KD pEPSCs at 48h post-transfection. Data represent the mean ± SD; n = 3 independent experiments; ns, non-significant; * *p* < 0.05; ** *p* < 0.01; two-tailed Student’s t test. C) Representative brightfield and tdTomato fluorescence morphology of *NANOG* KD and *POU5F1* KD pEPSCs at 48 h post-transfection. Scale bar, 100 μm. D) A differentiation culture system possesses temporal changes in pluripotency and lineage-specific gene expression during the differentiation process. E and F) Representative tdTomato fluorescence morphology (E) and flow cytometry profiles (F) showing the progressive reduction in tdTomato^+^ cell populations over time during the differentiation of *NANOG*-tdTomato and *POU5F1*-tdTomato cell lines. Scale bar, 100 μm.


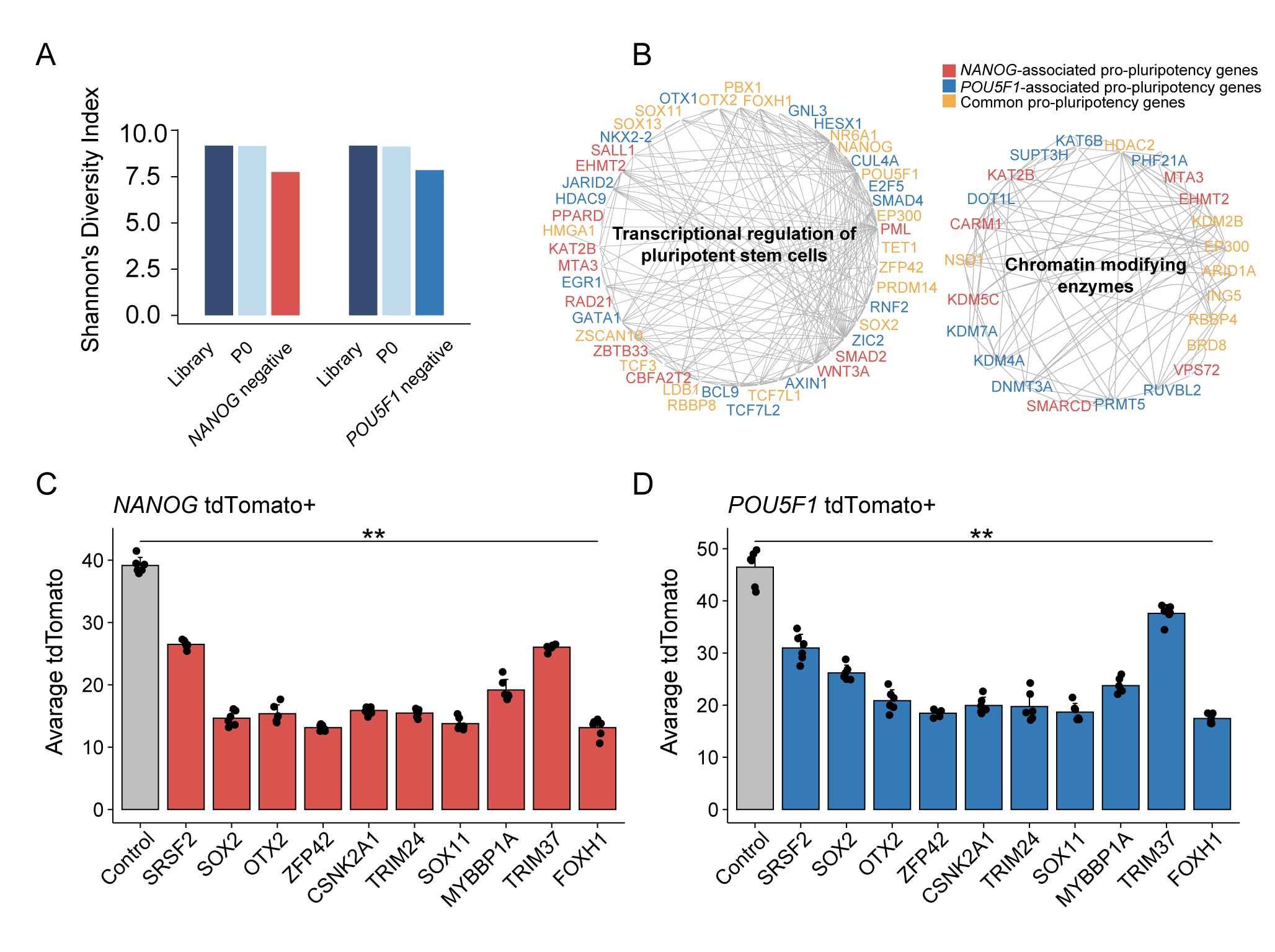


**Figure S4. Comprehensive analysis of pluripotency screening.** A) Shannon's Diversity Index comparing the sgRNA diversity within the initial plasmid library, the P0 mutant reporter cell library, and the mutant reporter-negative cell library for *NANOG*-tdTomato and *POU5F1*-tdTomato. B) Network diagram depicting the complex protein-protein interactions among potential candidate pluripotency genes associated with the transcriptional regulation of pluripotent stem cells and chromatin-modifying enzymes. C and D) Quantitative analysis of tdTomato fluorescence in *NANOG*-tdTomato cells (C) and *POU5F1*-tdTomato cells (D) after knockout of 10 candidate genes. ** *p* < 0.01; two-tailed Student’s t test.


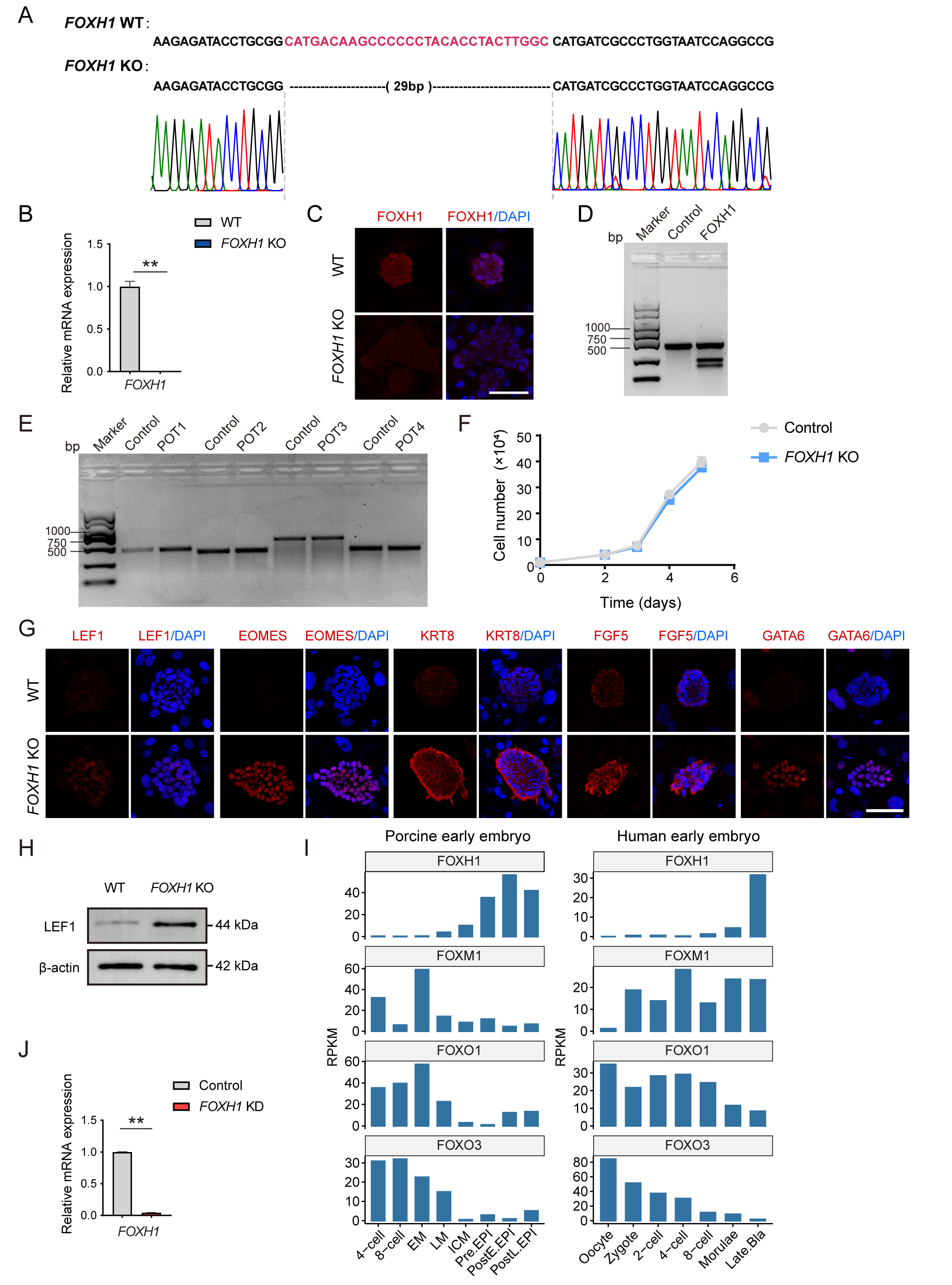


**Figure S5. Validation of *FOXH1* knockout and its impact on pluripotency.** A) Sequence alignment showing the deletion in the *FOXH1* gene in KO cells compared to WT. B) RT-qPCR analysis of *FOXH1* expression level in KO cells compared to WT. Data are shown as mean ± SD. ** *p* < 0.01; two-tailed Student’s t test. C) Immunofluorescence staining for FOXH1 (red) and nuclei (DAPI, blue) in WT and *FOXH1* KO pEPSCs. Scale bar, 100 μm. D and E) Detection of the *FOXH1* site (D) and four potential off-targets (E) using the T7ENⅠ cleavage assay. POT: Potential off-target effect; Control: WT cells; bp: base pairs. F) The proliferation rates of non-target control and *FOXH1* KO pEPSCs. G) Immunofluorescence staining for three germ layers markers in WT and *FOXH1* KO pEPSCs. Scale bar, 100 μm. H) Western blot analysis of LEF1, as one of the markers for the three germ layers, in WT and *FOXH1* KO pEPSCs, utilizing β-actin as an internal control for normalization. I) *FOXH1* expression in early porcine and human embryo development. J) RT-qPCR analysis of *FOXH1* expression level in negative control and *FOXH1* KD embryos. Data are shown as mean ± SD. ** *p* < 0.01; two-tailed Student’s t test.


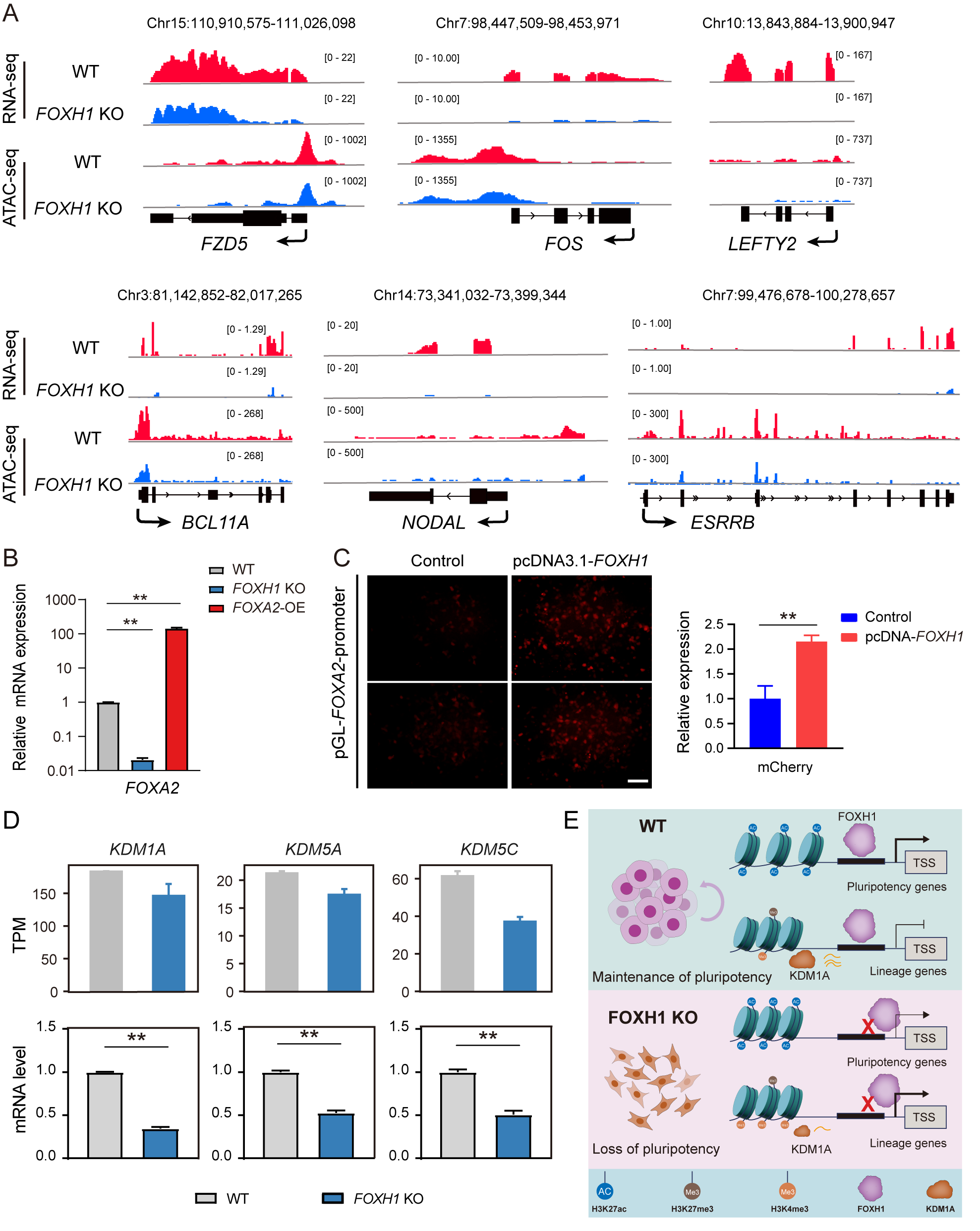


**Figure S6. Influence of FOXH1 on chromatin accessibility and regulation of its target genes.** A) The IGV snapshots show ATAC-seq and RNA-seq of the CRE regions of FOXH1 target pluripotency genes (*FZD5*, *FOS*, *LEFTY2* and others) in WT and *FOXH1* KO pEPSCs. B) RT-qPCR analysis of *FOXA2* expression in WT, *FOXH1* KO and overexpressed *FOXA2* based on *FOXH1* KO cells. Data are shown as mean ± SD. ** *p* < 0.01; two-tailed Student’s t test. C) Representative images of HEK293T cells co-transfected with empty vector or FOXH1 overexpression vector and FOXA2-promoter-mCherry reporter vector. Images from two independent experiments are shown. Scale bars, 100 μm. Bottom panel: Quantification of mCherry expression. Data are shown as mean ± SD. ** *p* < 0.01; two-tailed Student’s t test. D) Quantification of expression levels of *KDM1A*, *KDM5A* and *KDM5C* in WT and *FOXH1* KO cells is presented with RNA-seq data shown above and RT-qPCR data shown below. Data are shown as mean ± SD. ** *p* < 0.01; two-tailed Student’s t test. E) A working model for FOXH1 regulating the pluripotency maintenance of pEPSCs. Under undifferentiated pEPSC states, FOXH1 maintains an open chromatin structure of its target pluripotency-associated genes. Additionally, FOXH1 regulates the expression of KDM1A to sustain bivalent modifications at developmental genes, thereby preventing their premature expression. In the absence of FOXH1, diminished KDM1A expression in the promoter regions of specific developmental genes leads to increased enrichment of H3K4me3. This change facilitates the mRNA expression levels of these developmental genes, ultimately driving the differentiation of pEPSCs.

**Supplementary Tables**

Table S1. Derivation of pEPSCs from Bama pig preimplantation embryos, related to Figures S1.

Table S2. Feature of the porcine CRISPR/Cas9 knockout library, related to Figures 1 and 3.

Table S3. Essential genes of porcine/human/mouse ESC, related to Figure 2.

Table S4. Sequences of cDNA amplification primers, overexpression vector construction primers, quantitative PCR primers, and PCR detection primers, related to Figures 3-6.

Table S5. RNA-seq, ChIP-seq and ATAC-seq experiment summary, related to Figure 5.
